# Disease characteristics and outcomes of Croatian pediatric patients with acute lymphoblastic leukemia: pretreatment immunophenotypic predictors of high bone marrow minimal residual disease on day 15 of treatment

**DOI:** 10.3325/cmj.2025.66.100

**Published:** 2025-04

**Authors:** Klara Dubravčić, Ružica Lasan Trčić, Ernest Bilić, Josip Konja, Ljubica Rajić, Ranka Femenić, Maja Pavlović, Koraljka Gjadrov Kuveždić, Sunčica Ries, Mirna Sučić, Ivana Franić Šimić, Renata Zadro, Margareta Radić Antolic, Ivana Horvat, Gordana Jakovljević, Jelena Roganović, Srđana Čulić, Dubravka Kuljiš, Višnja Armanda, Karolina Malić Tudor, Senada Šerifi, Bernarda Lozić, Drago Batinić

**Affiliations:** 1 Division of Flow Cytometry, Department of Laboratory Diagnostics, Children's Hospital Zagreb, Zagreb, Croatia; 2 Division for Cytogenetics, Department of Laboratory Diagnostics, University Hospital Centre Zagreb, Zagreb, Croatia; 3 Department of Pediatric Hematology and Oncology, University Hospital Centre Zagreb, Zagreb, Croatia; 4 School of Medicine, University of Zagreb, Zagreb, Croatia; 5 Department of Pathology and Cytology, University Hospital Centre Zagreb, Zagreb, Croatia; 6 Division of Cytology, Department of Pathology and Cytology, Sveti Duh University Hospital, Zagreb, Croatia; 7 Department of Laboratory Diagnostics, St Catherine Specialty Hospital, Zagreb, Croatia; 8 Division of Laboratory Hematology and Coagulation, Department of Laboratory Diagnostics, University Hospital Centre Zagreb, Zagreb, Croatia; 9 Medical- biochemical Laboratory, Polyclinic Analiza, Varaždin, Croatia; 10 Department of Oncology and Hematology, Children's Hospital Zagreb, Zagreb, Croatia; 11 Faculty of Biotechnology and Drug Development, University of Rijeka, Rijeka, Croatia; 12 Department of Pediatrics, University Hospital of Split, Split, Croatia; 13 Department of Pediatric Hematology and Oncology, Department of Pediatrics, University Hospital Center Rijeka, Rijeka, Croatia; 14 University of Split School of Medicine, Split, Croatia; 15 Division of Laboratory Immunology, Department of Laboratory Diagnostics, University Hospital Centre Zagreb, Zagreb, Croatia; 15 School of Medicine, University of Zagreb, Zagreb, Croatia; * The author died on November 9, 2024, prior to article publication.

## Abstract

**Aim:**

To assess the clinical-biological characteristics and outcomes of Croatian pediatric patients with acute lymphoblastic leukemia (ALL). A secondary aim was to evaluate the predictive value of pretreatment leukemia-associated immunophenotypes (LAIPs) for poor early response to induction therapy defined as ≥10% day 15 bone marrow flow cytometry minimal residual disease (FCM-MRD).

**Methods:**

This retrospective cohort study reviewed the medical data of 393 consecutive pediatric ALL patients diagnosed and treated from February 2003 to April 2017 at four Croatian pediatric hemato-oncology centers. FCM data of 379 non-infant patients enrolled in two consecutive intercontinental trials, ALL IC-BFM 2002 (NCT00764907) and ALL IC-BFM 2009 (EudraCT 2010-019722-13), were analyzed to evaluate the association between LAIPs at diagnosis and day 15 FCM-MRD≥10% using univariate and multivariate logistic regression.

**Results:**

The median age at diagnosis was 5.2 years, with a predominance (83%) of B-cell precursor (BCP) ALL, and high hyperdiploidy (25.1%) and *ETV6::RUNX1* (18.7%) as the most common genetic abnormalities. The protocols did not significantly differ in 5-year event-free survival (82.1% vs 81.7%), overall survival (88% vs 85%), and cumulative incidence of relapse (12.3% vs 10%). FCM-MRD≥10% on day 15 was identified in 22.1% of patients and was predicted by white blood cell (WBC) count ≥20 × 10^9^/L (*P* = 0.011) and strong expression of CD34 (*P* = 0.032) and CD13 (*P* = 0.001) at diagnosis.

**Conclusion:**

The characteristics and survival rates of Croatian pediatric ALL patients aligned with ALL IC-BFM data. WBC≥20 × 10^9^/L, CD34^strong^, and CD13^strong^ independently predicted poor early response in BCP-ALL, suggesting a potential prognostic value of LAIPs at diagnosis.

Acute lymphoblastic leukemia (ALL) is the most common pediatric cancer, affecting children of all ages, with a peak incidence between two and five years of age and a slightly higher prevalence in boys (1). ALL is a heterogeneous disease originating from B- or T-cell precursors. Consequently, the contemporary multidisciplinary approach to leukemia diagnosis and classification by the World Health Organization (WHO) incorporates immunophenotype (lineage) and genetic abnormalities to define clinically, biologically, and therapeutically relevant disease entities (2).

The survival of children with ALL has improved dramatically, which represents one of the greatest accomplishments in oncology. This improvement is attributed to the progressive development of intensive multiagent chemotherapy protocols based on the results of international cooperative group trials and the introduction of risk-adapted therapy using pretreatment clinical-biological prognostic features and early treatment response assessment for more individually tailored therapy intensity (1,3).

Flow cytometry (FCM) is a highly reliable method for assessing leukemic lymphoblast clearance. Patients with high minimal residual disease (FCM-MRD)≥10% in bone marrow on day 15 during induction therapy fared particularly poorly, independent of initial clinical and biological features (4). A more sensitive real-time quantitative polymerase chain reaction (RQ-PCR) is better suited for assessing the quality of remission at later time points (4-7). Many countries within the International Berlin–Frankfurt–Münster Study Group (I-BFM-SG) do not perform PCR-MRD. National groups that joined the intercontinental consortium (ALL IC) agreed to apply only FCM-MRD on day 15 in the randomized clinical trial ALL IC-BFM 2009 in addition to the previous ALL IC-BFM 2002 risk stratification scheme (8–11).

MRD is currently the most powerful independent prognostic factor in childhood ALL as a measure of drug resistance *in vivo* and, therefore, a surrogate marker of an individual response (12,13). For that reason, MRD as a surrogate endpoint for the early evaluation of the treatment efficacy can rapidly resolve the problem of stratifying patients with rare or recently identified genetic and immunophenotypic aberrations, revealing their prognosis (14).

Blasts in ALL show immunophenotypic aberrations, ie, leukemia-associated immunophenotypes (LAIPs), even within the subcategories defined by the European Group for the Immunological Characterization of Leukemias (EGIL) classification (15,16). Although EGIL classification is not of prognostic value, LAIP characterization at diagnosis has reestablished its prognostic significance, not only for FCM-MRD but also for defining new high-risk leukemia subtypes characterized by specific immunophenotypes with poor early response and high MRD levels during therapy (17-25). To date, no studies have explored the immunophenotypic predictors of poor early treatment response, defined as FCM-MRD≥10% on day 15. Furthermore, our study provides the first overview of the characteristics and treatment outcomes of Croatian pediatric ALL patients.

The aim of this retrospective cohort study was to establish the clinical-biological characteristics and survival outcomes of Croatian pediatric ALL patients treated according to protocols ALL IC-BFM 2002 (NCT00764907) and ALL IC-BFM 2009 (EudraCT 2010-019722-13). Additionally, we characterized LAIPs at diagnosis and evaluated their association with high FCM-MRD levels in bone marrow on day 15 of induction therapy to assess their predictive potential for poor response.

## PATIENTS AND METHODS

### Patients and treatment protocols

We retrospectively reviewed data from 393 consecutive, unselected patients aged ≤18 years with *de novo* ALL diagnosed between February 2003 and April 2017 at four Croatian pediatric hemato-oncology centers (University Hospital Centre Zagreb, Children's Hospital Zagreb, University Hospital of Split, University Hospital Center Rijeka). Two patients continued treatment abroad, and 12 were infants enrolled in the INTERFANT 99/06 protocols. The remaining 379 patients aged >1 year were treated according to one of two subsequent protocols: ALL IC-BFM 2002 (n = 196; 49.9%) or ALL IC-BFM 2009 (n = 183; 46.6%), depending on the time of diagnosis (10,11). Details of diagnostics and risk stratification are given in Supplemental Methods(Supplemental Methods). Written informed consent was obtained from the parents or legal guardians of the participants before the initiation of treatment. The study was approved by the Ethics Committee of the University Hospital Center Zagreb and the Ethics Committee of the Faculty of Pharmacy and Biochemistry, University of Zagreb.

### Immunophenotypic analysis

Flow cytometry immunophenotyping of bone marrow aspirates at diagnosis and during follow-up was performed centrally in the Croatian Reference Centre for Immunodiagnostics of Hematological and Immunological Diseases, University Hospital Center Zagreb. Diagnostic bone marrow samples were collected before treatment and processed within 24 hours according to the standard procedure elaborated in Supplemental Methods(Supplemental Methods). LAIPs of leukemic blasts at diagnosis were determined and reported for each antigen according to AIEOP-BFM Consensus Guidelines 2016 for ALL immunophenotyping (16) (Supplemental Methods(Supplemental Methods)).

### Minimal residual disease assessment by flow cytometry

FCM-MRD was measured in bone marrow on day 15 of induction therapy after 14 days of prednisone, vincristine, daunorubicin, asparaginase, and intrathecal methotrexate. All samples were processed according to stain/lyse protocol, using BD FACS^TM^ Lysing Solution and analyzed using a FacsCalibur flow cytometer and Paint-a-Gate^TM^ software (all from BD Biosciences, San Jose, CA, USA), as previously described (9,26). LAIPs of leukemic lymphoblasts were identified at diagnosis as deviations from normal lymphocyte differentiation/maturation patterns defined by previously published immunophenotypes of hematogones (27). For this purpose, the following monoclonal fluorochrome-conjugated antibody combinations were used: 1) SYTO16/CD10-PE/CD45-PerCP/CD19-APC; 2) CD20-FITC/CD10-PE/CD34-PerCP-Cy5.5/CD19-APC; 3) CD58-FITC/CD10-PE/CD45-PerCP/CD19-APC; and 4) CD10 + 20-FITC/CD38-PE/CD45-PerCP/CD19-APC for BCP-ALL; and 1) SYTO16/CD7-PE/CD45-PerCP/CD3-APC; 2) CD99-FITC/CD7-PE/CD5-PerCP-Cy5.5/CD3-APC; 3) TdT-FITC/CD7-PE/CD3-PerCP/citCD3-APC; and 4) CD4-FITC/CD8-PE/CD45-PerCP/CD3-APC for T-ALL. The same panels were used during the follow-up for FCM-MRD detection. The presence of nonnucleated cells (platelets, red cell fragments, and debris) and hemodilution was corrected using the cell-permeant nucleic acid-binding dye SYTO16. A total of 300 000 nucleated (SYTO16+) events were acquired. Samples with significant hemodilution, defined as having less than 2% erythroblasts (SYTO16+CD45-lineage-) or fewer than 50% SYTO16+ events, were excluded from patient stratification. FCM-MRD is quantified as the percentage of leukemic cells among nucleated (SYTO16+) cells.

### Cytogenetic and molecular analysis

Conventional cytogenetic and fluorescence *in situ* hybridization (FISH) with commercially available probe sets were performed routinely on bone marrow samples prior to therapy (28). Karyotypes were described according to the International System of Human Cytogenetic Nomenclature (ISCN, 2009) (29). The presence of *BCR::ABL1, KMT2A::AFF1, ETV6::RUNX1*, and *TCF3::PBX1* fusion gene transcripts was examined as part of routine ALL molecular diagnostics using standardized reverse transcription-polymerase chain reaction (RT-PCR) analysis, following the BIOMED-1 protocol (30).

### Statistical analysis

Differences between patient groups were assessed with χ^2^ or Fisher exact tests for categorical variables and the Mann-Whitney U test for continuous variables. Statistical details are provided in Supplemental Methods(Supplemental Methods). The primary endpoint of the study was the day 15 FCM-MRD in bone marrow, categorized as a dichotomous variable (FCM-MRD<10% vs FCM-MRD≥10%). Logistic regression was conducted to identify LAIP and other independent predictors of FCM-MRD≥10%. Associations between FCM-MRD and assessed variables were expressed as odds ratios (OR) with 95% confidence intervals (CI). Receiver operating characteristic (ROC) curves and the area under the curve (AUC) were used to assess the discriminatory ability of predictors. The Kaplan-Meier method with log-rank tests for group comparisons was used to estimate survival rates. Event-free survival (EFS) and overall survival (OS) were calculated from the date of diagnosis to relapse, resistance, second malignant neoplasm, and/or death alone. The cumulative incidence of relapse (CIR) was compared between groups using a Gray's test, considering death before relapse as a competing risk. Patients’ follow-up data were updated in June 2023. Two-tailed *P* < 0.05 was considered statistically significant. Statistical analyses were performed with SPSS, version 26 (IBM, Armonk, NY, USA), and SAS OnDemand for Academics (SAS Institute Inc., Cary, NC, USA).

## RESULTS

### Clinical and biological characteristics of Croatian pediatric ALL patients

The median age at diagnosis was 5.2 years (range, 0-17.5 years), with a predominance of boys (61.3%). The highest proportion of patients was in the age group 1-6 years (n = 214; 54.5%), while the lowest was in the age group 16-18 years (n = 15; 3.8%) and in the infant group (n = 12; 3.1%) (Table 1, Supplemental Table 1(Supplemental Table 1), and Supplemental Table 2(Supplemental Table 2)).

**Table 1:** Patients’ characteristics according to immunophenotype (BCP-ALL vs T-ALL)*

		Total	BCP-ALL	T-ALL	P^†^
n (%)		393 (100.0)	326 (83.0)	67 (17.0)	
		n (%)	n (%)	n (%)	
**Sex**					0.057
male		241 (61.3)	193 (59.2)	48 (71.6)	
female		152 (38.7)	133 (40.8)	19 (28.4)	
**Age** (years)					
median (range)		5.2 (0.0–17.5)	4.6 (0.0–17.5)	9.2 (1.6–17.3)	<0.001^‡^
<1		12 (3.1)	12 (3.7)	0 (0.0)	<0.001
≥1 –<6		214 (54.5)	193 (59.2)	21 (31.3)	
≥6 –<10		62 (15.8)	49 (15.0)	13 (19.4)	
≥10 –<16		90 (22.9)	64 (19.6)	26 (38.8)	
≥16 –<18		15 (3.8)	8 (2.5)	7 (10.4)	
**WBC count** ( × 10^9^/L)					<0.001
<20		234 (60.6)	222 (69.4)	12 (18.2)	
≥20		152 (39.4)	98 (30.6)	54 (81.8)	
no information		7	6	1	
**CNS status**					0.109
CNS1		355 (92.2)	299 (93.1)	56 (87.5)	
CNS2		20 (5.2)	16 (5.0)	4 (6.3)	
CNS3		10 (2.6)	6 (1.9)	4 (6.3)	
No information		8	5	3	
**Splenomegaly**					0.006
no		188 (49.3)	166 (52.5)	22 (33.8)	
yes		193 (50.7)	150 (47.5)	43 (66.2)	
no information		12	10	2	
**Hepatomegaly**					0.657
no		150 (39.4)	126 (39.9)	24 (36.9)	
yes		231 (60.6)	190 (60.1)	41 (63.1)	
no information		12	10	2	
**Mediastinal mass**					<0.001
no		340 (90.4)	309 (99.0)	31 (48.4)	
yes		36 (9.6)	3 (1.0)	33 (51.6)	
no information		17	14	3	
**Testicular involvement**					0.347
no		233 (99.1)	189 (99.5)	44 (97.8)	
yes		2 (0.9)	1 (0.5)	1 (2.2)	
no information		6	3	3	
**Genetic prognostic groups**					<0.001
favorable		105 (27.6)	104 (32.5)	1 (1.6)	
intermediate		245 (64.3)	188 (58.8)	57 (93.4)	
poor		31 (8.1)	28 (8.8)	3 (4.9)	
no information		12	6	6	
**Relapse**					0.338
no		340 (87.0)	285 (87.7)	55 (83.3)	
yes		51 (13.0)	40 (12.3)	11 (16.7)	
no information		2	1	1	
**Site of relapse**					0.095
isolated BM		33 (64.7)	28 (70.0)	5 (45.5)	
isolated CNS		8 (15.7)	4 (10.0)	4 (36.4)	
isolated testicular		2 (3.9)	1 (2.5)	1 (9.1)	
combined		8 (15.7)	7 (17.5)	1 (9.1)	
**Time to relapse** (months from diagnosis)					0.006
very early (less than 18)	20 (39.2)		11 (27.5)	9 (81.8)	
early (18-30)	11 (21.6)		11 (27.5)	0 (0.0)	
late (more than 30)	20 (39.2)		18 (45.0)	2 (18.2)	
**Death**					0.186
no	331 (84.4)		278 (85.5)	53 (79.1)	
yes	61 (15.6)		47 (14.5)	14 (20.9)	
no information	1		1	0	
**HSCT**					
matched related donor (brother/sister)	22 (54.7)		13 (46.4)	9 (69.2)	
mismatched related donor	1 (2.4)		0 (0.0)	1 (7.7)	
matched unrelated donor	18 (43.9)		15 (53.6)	3 (23.1)	
**Protocols**					
ALL IC-BFM 2002	196 (49.9)		150 (46.0)	46 (68.7)	
ALL IC-BFM 2009	183 (46.6)		162 (49.7)	21 (31.3)	
INTERFANT 99/06	12 (3.1)		12 (3.7)	0 (0.0)	
Continued treatment abroad	2 (0.5)		2 (0.6)	0 (0.0)	

*Abbreviations: ALL – acute lymphoblastic leukemia; BCP – B-cell precursor; BM – bone marrow; CNS – central nervous system; HSCT – hematopoietic stem cell transplantation; WBC – white blood cells.

†χ^2^ or Monte Carlo simulated Fisher exact test comparing BCP- and T-ALL groups; patients with no information were excluded from the analysis.

‡Mann-Whitney U test.

**Figure 1:**
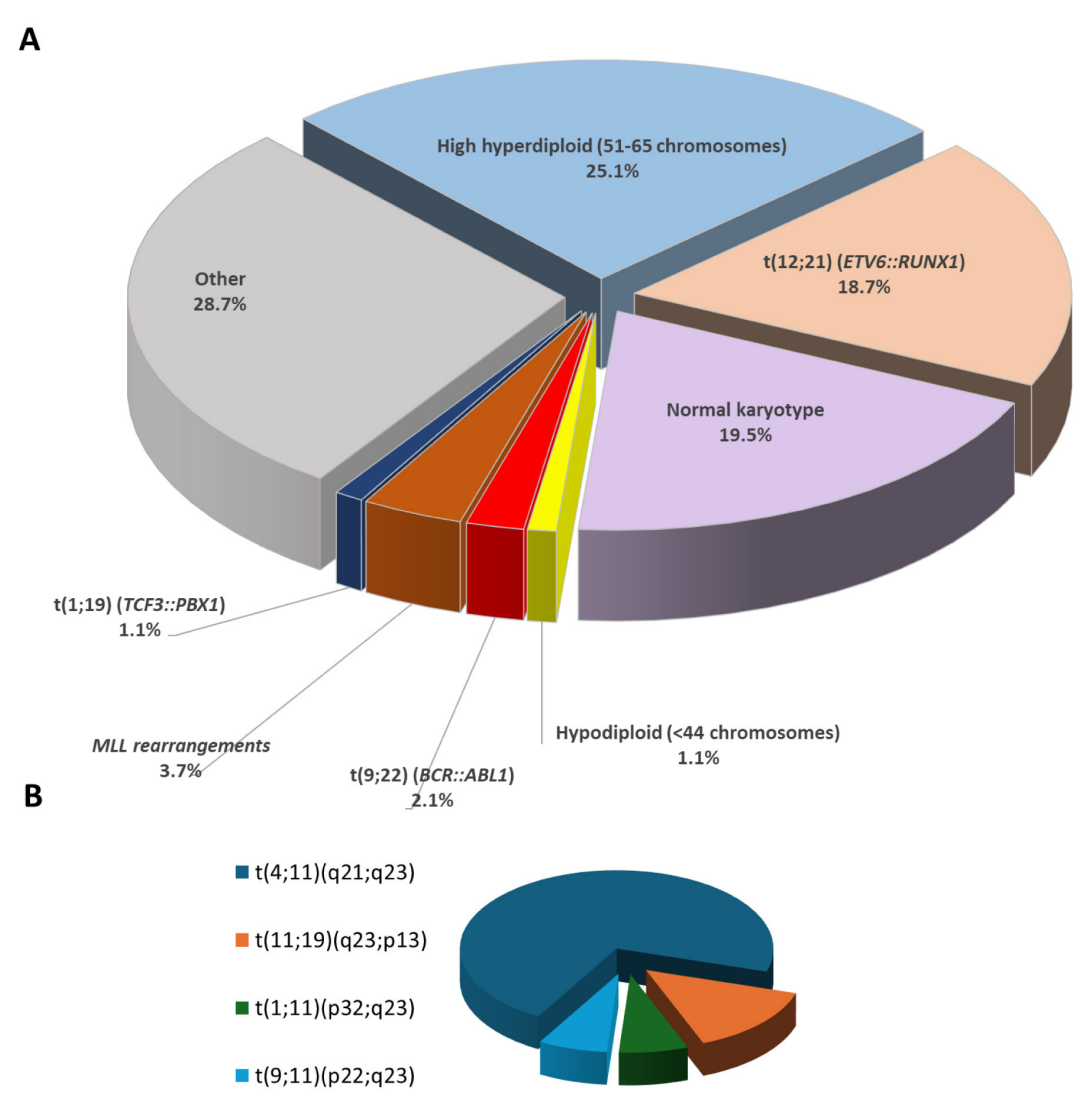
The frequency of major cytogenetic subgroups in Croatian children with acute lymphoblastic leukemia (ALL), shown for (**A**) the cohort with available cytogenetic results (N = 374) and (**B**) patients with mixed-lineage leukemia (MLL) rearrangements (N = 14).

Conventional cytogenetics was successfully performed in 374 patients (95.2%). Normal karyotype was identified in 73 patients (19.5%). The most frequent recurrent abnormalities were high hyperdiploidy (51-65 chromosomes) in 94 (25.1%) and t (12;21) ETV6::RUNX1 in 70 (18.7%) patients, followed by mixed-lineage leukemia (MLL) gene rearrangements in 14 (3.7%), t (9;22) BCR::ABL1 in 8 (2.1%), t (1;19) *TCF3::PBX1* in 4 (1.1%), and hypodiploidy (<44 chromosomes) in 4 patients (1.1%). The “other” category (28.7%) comprises all the remaining cytogenetic abnormalities (Figure 1).

High hyperdiploidy and t (12;21) were predominantly found in the age group 1-6 years (*P* < 0.001), whereas *MLL* gene rearrangements were predominantly found in infants, representing 58.3% of all infant ALL cases (*P* < 0.001) (Supplemental Figure 1A(Supplemental Figure 1)).

According to cytogenetic and molecular criteria, patients were categorized into three prognostic groups: favorable (high hyperdiploidy and t (12;21) without other abnormalities; n = 105; 27.6%), poor (MLL rearrangements, t (9;22), hypodiploidy, t (17;19), 17p abnormalities, and 13q loss; n = 31; 8.1%), and intermediate (all other genetic abnormalities; n = 245; 64.3%).

Most patients (n = 326; 83%) had B-cell precursor (BCP) ALL, while 67 patients (17%) had T-cell ALL (T-ALL). The latter were significantly older, had higher WBC, a higher frequency of splenomegaly and mediastinal masses, less favorable genetics, and a higher incidence of a very early relapse (Table 1; Supplemental Figure 2(Supplemental Figure 2)).

According to EGIL, most BCP-ALL cases were classified as the “common” subtype (B-II; n = 235; 72.1%), followed by pre-B (B-III; n = 76; 23.3%) and pro-B (B-I; n = 15; 4.6%) subtype. Pro-B patients were mostly infants (53%, *P* < 0.001) and had significantly higher WBC, CNS3 status, and poor prognostic genetic abnormalities (Supplemental Table 1(Supplemental Table 1)).

The “common” ALL was significantly associated with high hyperdiploidy (*P* < 0.001), and the pro-B immunophenotype with *MLL* gene rearrangements (*P* < 0.001), primarily t (4;11) (10 of 14 cases). Most patients with t (1;19) translocation (3 of 4) had a pre-B immunophenotype; however, this association did not reach statistical significance (Supplemental Figure 1B(Supplemental Figure 1)).

In T-ALL, the most common subtype was cortical T-ALL (T-III; n = 30; 44.7%), followed by pre-T (T-II; n = 16; 23.9%), mature T-ALL (T-IV; n = 12; 17.9%), and pro-T (T-I; n = 4; 6%). These patients did not differ in clinical or genetic characteristics at diagnosis, except for a significant association between mediastinal mass and the cortical immunophenotype (Supplemental Table 2(Supplemental Table 2)).

### Treatment outcomes and risk group distribution

**Figure 2:**
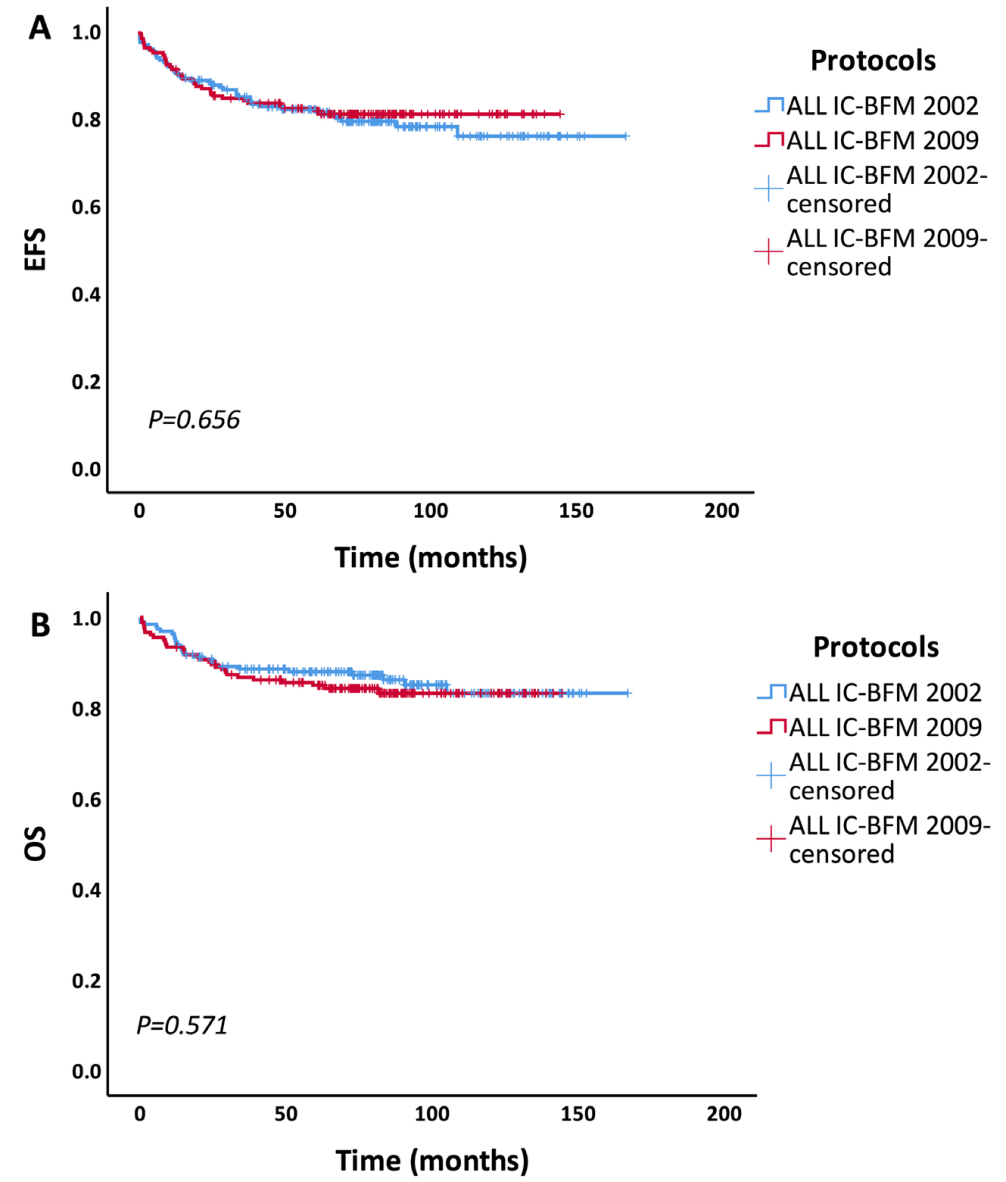
Kaplan-Meier survival curves for (**A**) event-free survival (EFS) and (**B**) overall survival (OS) in non-infant patients (aged 1–18 years) treated according to the ALL IC-BFM 2002 and ALL IC-BFM 2009 protocols. ALL – acute lymphoblastic leukemia; IC-BFM – international consortium Berlin–Frankfurt–Münster.

**Figure 3:**
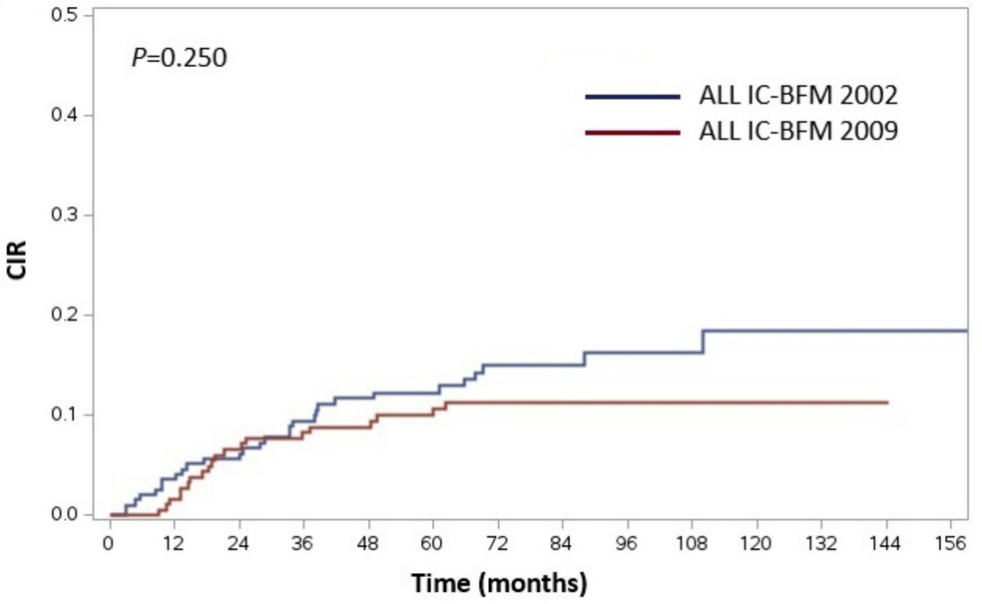
Cumulative incidence of relapse (CIR) in non-infant patients (aged 1–18 years) treated according to the ALL IC-BFM 2002 and ALL IC-BFM 2009 protocols. ALL – acute lymphoblastic leukemia; IC-BFM – international consortium Berlin–Frankfurt–Münster.

Of 379 non-infant patients, 196 (51.7%) were treated according to the ALL IC-BFM 2002 protocol and 183 (48.3%) according to the ALL IC-BFM 2009 protocol. The median follow-up was 80.3 months for ALL IC-BFM 2002 and 76.8 months for ALL IC-BFM 2009. Forty-nine patients relapsed (13%) and 56 patients (14.8%) died (Table 2). No significant differences in survival were observed between the protocols. The 5-year event-free survival (EFS ± SE) was 82.1% ± 2.8% for ALL IC-BFM 2002 and 81.7% ± 2.9% for ALL IC-BFM 2009 (*P* = 0.656). The 5-year overall survival (OS ± SE) was 88.0% ± 2.3% and 85.0% ± 2.7%, respectively (*P* = 0.571) (Figures 2A and 2B). The 5-year cumulative incidence of relapse (CIR ± SE) was higher in ALL IC-BFM 2002 but not significantly so (12.3% ± 2.4% vs 10% ± 2.3%; *P* = 0.250) (Figure 3). Relapses were classified as very early (within 18 months from diagnosis; 38.8%), early (18-30 months; 20.4%), and late (>30 months from diagnosis; 40.8%) (Table 2).

**Table 2:** Early response characteristics, risk group distribution, and outcomes of patients treated with the ALL IC-BFM 2002 and ALL IC-BFM 2009 protocols*

		Protocols		
	Total	ALL IC-BFM 2002	ALL IC-BFM 2009	P†
n (%)	379 (100.0)	196 (51.7)	183 (48.3)	
	n (%)	n (%)	n (%)	
**Prednisone response day 8**				0.087
GPR	344 (93.2)	173 (91.1)	171 (95.5)	
PPR	25 (6.8)	17 (8.9)	8 (4.5)	
no information	10	6	4	
**BM morphology day 15**				0.014
M1	266 (77.6)	134 (72.0)	132 (84.1)	
M2	54 (15.7)	34 (18.3)	20 (12.7)	
M3	23 (6.7)	18 (9.7)	5 (3.2)	
no information	36	10	26	
**FCM-MRD day 15**				
MRD<0.1%		n/a	38 (27.5)	
MRD 0.1%-10%		n/a	68 (49.3)	
MRD≥10%		n/a	32 (23.2)	
no information/inadequate sample			45	
**FCM-MRD day 15**				0.692
MRD<10%	239 (77.9)	133 (78.7)	106 (76.8)	
MRD≥10%	68 (22.1)	36 (21.3)	32 (23.2)	
no information/inadequate sample	72	27	45	
**Risk group**				<0.001
SR	82 (21.8)	60 (30.9)	22 (12.1)	
IR	218 (58.0)	98 (50.5)	120 (65.9)	
HR	76 (20.2)	36 (18.6)	40 (22.0)	
not stratified^‡^	3	2	1	
**Relapse**				
no	328 (87.0)	167 (85.2)	161 (89.0)	0.280
yes	49 (13.0)	29 (14.8)	20 (11.0)	
**Time to relapse** (months from diagnosis)				0.290
very early (less than 18)	19 (38.8)	11 (37.9)	8 (40.0)	
early (18–30)	10 (20.4)	4 (13.8)	6 (30.0)	
late (more than 30)	20 (40.8)	14 (48.3)	6 (30.0)	
**Death**				0.570
no	323 (85.2)	169 (86.2)	154 (84.2)	
yes	56 (14.8)	27 (13.8)	29 (15.8)	

*Abbreviations: ALL – acute lymphoblastic leukemia; BM – bone marrow; FCM – flow cytometry; GPR – good prednisone response (<1000 blasts/μL in peripheral blood on day 8); HR – high risk; IC-BFM – international consortium Berlin–Frankfurt–Münster; IR – intermediate risk; M1 –<5% blasts; M2 – 5–25% blasts; M3 –≥25% blasts in bone marrow; MRD – minimal residual disease; PPR – poor prednisone response (≥1000 blasts/μL in peripheral blood on day 8); SR – standard risk.

†χ^2^ test; patients without data or with an inadequate sample were excluded from the analysis.

‡Patients died during or immediately after the induction phase of treatment.

In the ALL IC-BFM 2002 protocol, patients were classified into risk groups as follows: 60 (30.9%) in the standard-risk, 98 (50.5%) in the intermediate-risk, and 36 (18.6%) in the high-risk group (Table 2). Survival outcomes differed significantly among all risk groups (*P* < 0.001 for both EFS and OS): the 5-year EFS and OS were 92.8% ± 3.5% and 98.0% ± 2.0% in the standard-risk group; 86.1% ± 3.6% and 92.8% ± 2.6% in the intermediate-risk; and 57.8% ± 8.3% and 62.9% ± 8.2% in the high-risk group, respectively (Supplemental Figures 3A and 3B(Supplemental Figure 3)). Among the three risk groups, the 5-year CIR values were 7.2% ± 3.5%, 9.8% ± 3.1%, and 28.3% ± 7.7%, respectively (*P* = 0.004). Pairwise comparisons of CIR revealed significant differences between the standard and high-risk group (*P* = 0.001) and between the intermediate and high-risk group (*P* = 0.022), but not between the standard and intermediate-risk group (*P* = 0.148) (Supplemental Figure 4A(Supplemental Figure 4)).

Day 15 FCM-MRD was introduced as an additional risk stratification criterion in the ALL IC-BFM 2009 protocol, which resulted in a significant reallocation of patients across risk groups: 22 (12.1%) in the standard-risk, 120 (65.9%) in the intermediate-risk, and 40 (22.0%) in the high-risk group (*P* < 0.001) (Table 2). The EFS, OS, and CIR were 95.5% ± 4.4%, 95.0% ± 4.9%, and 4.6% ± 4.6% in the standard-risk; 84.6% ± 3.3%, 89.0% ± 2.9%, and 8.7% ± 2.7% in the intermediate-risk; and 67.5% ± 7.4%, 70.0% ± 7.2%, and 20% ± 6.4% in the high-risk group, respectively. Survival differed significantly between risk groups, except between the standard-risk and intermediate-risk group for EFS (*P* = 0.167) and OS (*P* = 0.264) (Supplemental Figures 3C and 3D(Supplemental Figure 3)). Pairwise comparison of CIR showed no significant differences between the high-risk group and the standard-risk group (*P* = 0.092) or the intermediate-risk group (*P* = 0.052) (Supplemental Figure 4B(Supplemental Figure 4)).

### Association of day 15 FCM-MRD≥10% with patients’ characteristics

Of the 379 non-infant patients enrolled in the ALL IC-BFM 2002/2009 protocols, 72 (19%) lacked FCM-MRD data. Among the remaining patients, 68 (22.1%) had FCM-MRD≥10% (Supplemental Table 3(Supplemental Table 3)). FCM-MRD≥10% was significantly associated with older age, higher WBC at diagnosis, and relapse, in BCP-ALL but not in T-ALL. Regarding the cytomorphological response to therapy, FCM-MRD≥10% was significantly associated with poor prednisone response (PPR) on day 8 and M2/M3 bone marrow on day 15 in both BCP- and T-ALL (Supplemental Table 4(Supplemental Table 4)).

### Association of day 15 FCM-MRD≥10% with LAIPs

FCM-MRD≥10% levels did not differ between BCP- and T-ALL or among the EGIL immunophenotypic categories, although PPR and M3 bone marrow were significantly associated with T-ALL (Supplemental Table 3(Supplemental Table 3) and Supplemental Table 4(Supplemental Table 4)).

A preliminary analysis of the association between LAIPs and FCM-MRD levels is shown in Supplemental Table 5(Supplemental Table 5) and Supplemental Table 6(Supplemental Table 6). Due to the immunophenotypic heterogeneity of individual CD marker expression and the limited number of events within LAIP categories, most expression categories in BCP-ALL were pooled into two clusters: negative/weak (negative/dim/partially positive 1) and strong (medium/heterogeneous/bright/partially positive 2). In BCP-ALL, FCM-MRD≥10% was significantly associated with CD19^dim^, CD34^strong^, CD45^strong^, and the myeloid marker CD13^strong^ (Supplemental Table 5(Supplemental Table 5)).

To identify predictive indicators of poor response to treatment, multivariate logistic regression included all significant pre-treatment variables from the univariate analysis: age, WBC count, CD13, CD19, CD34, and CD45, along with sex and genetic prognostic groups, both of which were at the limit of statistical significance (*P* = 0.065 and *P* = 0.062, respectively) (Table 3). After adjustment, WBC≥20 × 10^9^/L (OR 2.870; 95% CI 1.276–6.456; *P* = 0.011), CD13^strong^ (OR 5.005; 95% CI 1.888–13.272; *P* = 0.001), and CD34^strong^ (OR 4.183; 95% CI 1.129–15.500; *P* = 0.032) remained significant and independent predictors of day 15 FCM-MRD≥10%. The combined score of these three predictors demonstrated good discriminatory power (AUC 0.728; 95% CI: 0.647–0.809) (Supplemental Figure 5A(Supplemental Figure 5)). These predictors retained their significance even after excluding patients from the poor genetic prognostic group (Supplemental Table 7(Supplemental Table 7)), with a slightly higher AUC of 0.734 (95% CI: 0.651–0.817) (Supplemental Figure 5B(Supplemental Figure 5)).

**Table 3:** Univariate and multivariate logistic regression analyses for predictors of high FCM-MRD (≥10%) on day 15 in BCP-ALL patients treated with ALL IC-BFM 2002/2009 protocols*

**Variable**	**Univariate logistic regression**			**Multivariate logistic regression**		
	**OR**	**95% CI**	**P**	**OR**	**95% CI**	**P**
Male sex	1.869	0.962–3.629	0.065	1.770	0.782–4.005	0.170
Age ≥6 years	2.226	1.193–4.155	0.012	1.294	0.530–3.159	0.572
WBC≥20x10^9^/L	2.809	1.490–5.298	0.001	2.870	1.276–6.456	0.011
EGIL subtype						
pro-B (B-I)	Reference					
common (B-II)	0.282	0.017–4.603	0.374			
pre-B (B-III)	0.157	0.009–2.767	0.206			
Genetic prognostic group						
favorable	Reference			Reference		
intermediate	1.092	0.553–2.156	0.800	1.046	0.455–2.401	0.916
poor	3.042	0.948–9.762	0.062	1.756	0.282–10.934	0.546
Antigen						
CD10 N/weak	1.313	0.134–12.896	0.815			
CD13 strong	3.681	1.636–8.280	0.002	5.005	1.888–13.272	0.001
CD19 weak	5.589	1.210–25.819	0.028	3.810	0.498–29.141	0.198
CD20 strong	0.993	0.505–1.953	0.983			
CD33 strong	1.371	0.631–2.980	0.425			
CD34 strong	5.848	1.747–19.575	0.004	4.183	1.129–15.500	0.032
CD45 strong	4.244	1.302–13.834	0.017	2.816	0.639–12.408	0.171
CD117 strong	1.347	0.134–13.530	0.800			
CD58 overexpressed	1.475	0.167–13.039	0.726			
TdT N/weak	0.520	0.149–1.818	0.306			

*Abbreviations: ALL – acute lymphoblastic leukemia; CI – confidence interval; EGIL – European Group for Immunological Classification of Leukemias; FCM-MRD – flow cytometry minimal residual disease IC-BFM – international consortium Berlin–Frankfurt–Münster N – negative; OR – odds ratio; WBC – white blood cells.

Blasts in T-ALL exhibited less heterogeneous immunophenotypic aberrations; therefore, the expression of their CD markers was reported qualitatively as negative or positive. A significant association was found between FCM-MRD≥10% and CD8^negative^, CD10^negative^, CD34^positive^, TdT^N/dim^, and the myeloid markers CD33^positive^ and CD117^positive^ (Supplemental Table 6(Supplemental Table 6)). Due to the small number of T-ALL patients and events, a multivariate analysis could not be performed.

## Discussion

Our study provides the first overview of the characteristics and outcomes of Croatian pediatric ALL patients diagnosed and treated between 2003 and 2017. The main findings confirmed a high predominance of BCP-ALL cases, a peak incidence in preschool age, and a slight male predominance, consistent with previous reports (1,31,32). The distribution of EGIL immunophenotypic subtypes, WHO-defined recurrent genetic aberrations, and their mutual association aligned with published data (2,33-37). Taken together, our findings support the reproducibility of the EGIL classification as a clinically relevant tool for diagnostics, characterization of ALL patient cohorts, and initial prognostic guidance (15,16).

The survival outcomes of ALL IC-BFM 2002 and ALL IC-BFM 2009 protocols in our cohort were comparable to those reported by major collaborative study groups worldwide (3,10,11,15,38-40).

In alignment with the overall results of the intercontinental ALL IC-BFM study group (11), the implementation of standardized FCM-MRD detection on day 15 in ALL IC-BFM 2009 led to significant differences in risk-group distribution, however without substantial differences in the outcomes between the two protocols, probably due to similar treatment intensity in both studies. Our CIR rates were slightly lower in the ALL IC-BFM 2009 protocol (10% vs 12.3%), particularly in the standard-risk (4.6% vs 7.2%) and high-risk group (20% vs 28.3%). This reduction likely reflects the impact of FCM-MRD introduction on risk stratification and, subsequently, stricter risk group assignments (which led to the reallocation of patients from the standard-risk group to the intermediate-risk group) and advancements in supportive care. In this context, when comparing FCM-MRD and morphology on day 15, we found some discrepant cases in which 18 M1 patients had FCM-MRD≥10% (data not shown). This indicates that morphological findings should be interpreted with caution, particularly when assessment is not centralized (4).

MRD provides early insights into individual therapy response, reflecting the combined impact of patient characteristics, leukemia cell biology, and therapy-related factors on blast reduction (5,12,13). Given these findings and considering that immunophenotype reflects the intrinsic properties of leukemic cells, we explored the predictive potential of pretreatment LAIPs for poor early response. The AEIOP-BFM ALL 2000 study first demonstrated that LAIP-based FCM-MRD analysis in bone marrow on day 15 was the most powerful early predictor of relapse, with high FCM-MRD (≥10%) associated with unfavorable outcomes (4).

To our knowledge, this is the first study to investigate the association between diagnostic LAIPs and FCM-MRD≥10% on day 15 in bone marrow, showing that the strong CD13 and CD34 expression and WBC count ≥20 × 10^9^/L significantly predicts poor early treatment response in BCP-ALL, independent of other clinical and genetic risk factors.

Nowadays, LAIP analysis is a well-established method for FCM-MRD monitoring during treatment (6,8,9,17,18,26,41). Many studies have also investigated the prognostic significance of individual LAIP markers such as CD13 (42-45), CD20 (46-50), CD34 (51,52), or CD45 (23,46,53), but often with conflicting results due to cohort heterogeneity, treatment regimens, and variations in FCM methodology and data interpretation. In contrast, few studies have focused on the direct association between LAIPs at diagnosis and MRD levels. In 2022, Modvig et al were the first to demonstrate an association between a CD34^+^CD38^dim+^nTdT^dim+^ immunophenotype on leukemic blasts in Philadelphia (Ph)-negative BCP-ALL and poor therapy response, predicting a PCR-MRD≥10^−5^ at the end of induction (EOI) on day 29. Their study identified CD34^high^ as the most significant independent predictor of EOI MRD, associated with an overrepresentation of genes related to stemness, cell migration, adhesion, and negative regulation of apoptosis (54).

In our study, CD13 was the most significant predictor of FCM-MRD≥10% in the entire BCP-ALL cohort. However, after excluding the poor genetic group, which included eight Ph-positive t (9;22) BCP-ALL patients, CD34 emerged as the most robust independent predictor, consistent with the findings of Modvig et al (54). CD13 is an N-aminopeptidase found on myeloid (42,43,55) and hematopoietic stem cells (56) and is often overexpressed in various types of cancers (57). Saxena et al demonstrated that CD13 was transiently expressed on a small subset of highly proliferative early B-cell precursors, while extended CD13 expression and dysregulation may trigger leukemogenesis and inhibit apoptosis (55). In BCP-ALL, CD13 expression was associated with t (9;22) and t (12;21) translocations (33,58-60), but its prognostic value remained uncertain, with reports ranging from no significance (42) to poor prognosis in adults (43,44). One report involving 58 children with ALL identified high WBC and CD13 as significant predictors of poor early response on day 33 (61).

Altogether, the results mentioned above support the findings of our study. However, the retrospective design, relatively small and heterogeneous cohort, missing or incomplete data, and limited diagnostic panels of monoclonal antibodies may affect the generalizability of our findings. An important limitation of our study is the small number of T-ALL patients. As a result, analyses were limited to preliminary association tests, which identified a significant relationship between FCM-MRD≥10% and individual LAIPs (CD8^negative^, CD10^negative^, CD34^positive^, and the myeloid markers CD33^positive^ and CD117^positive^), many of which are part of the diagnostic criteria for early T-cell precursor-ALL characterized by poor early response (20,21).

In conclusion, our extensive retrospective study provides valuable insights into clinical-biological characteristics, early treatment response, and outcomes of Croatian pediatric ALL patients. The implementation of standardized FCM-MRD detection on day 15 in the ALL IC-BFM 2009 protocol led to more rigorous risk stratification, resulting in the reallocation of patients from the standard-risk to the intermediate-risk group, which in the future could be used for improved risk-based treatment. Furthermore, our findings underscore the potential of pretreatment immunophenotype as a predictor of poor early response in BCP-ALL. Our results warrant further validation in larger, well-designed prospective studies. Thus, understanding the leukemic cell properties could refine risk stratification, enhance treatment protocols, and advance personalized medicine strategies in pediatric ALL.
