# Supplementary material for: Disease characteristics and outcomes of Croatian pediatric patients with acute lymphoblastic leukemia: pretreatment immunophenotypic predictors of high bone marrow minimal residual disease on day 15 of treatment
Source: Croat Med J. 2025 Apr;66(2):100–14. doi: 10.3325/cmj.2025.66.100 (PMC12093125; doi:10.3325/cmj.2025.66.100)

SUPPLEMENTAL FIGURE 1. Distribution of cytogenetic abnormalities in the entire cohort of Croatian pediatric acute lymphoblastic leukemia (ALL) patients by (A) age groups and (B) EGIL subtypes. Abbreviations: EGIL – European Group for the Immunological Classification of Leukemias.

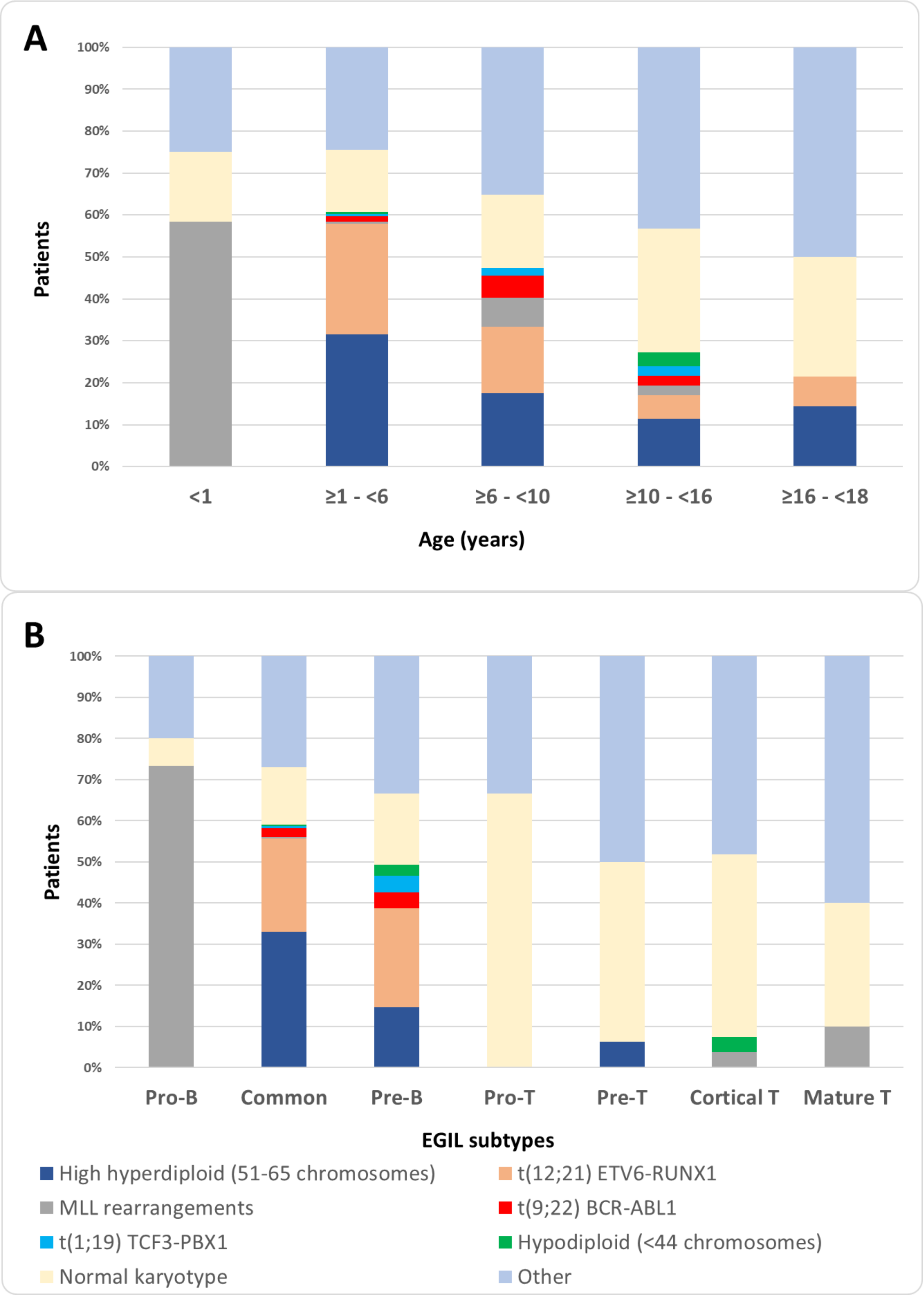

Supplement: Supplemental Figure 1 [file CroatMedJ_66_s001.pdf]
