# Supplementary material for: Disease characteristics and outcomes of Croatian pediatric patients with acute lymphoblastic leukemia: pretreatment immunophenotypic predictors of high bone marrow minimal residual disease on day 15 of treatment
Source: Croat Med J. 2025 Apr;66(2):100–14. doi: 10.3325/cmj.2025.66.100 (PMC12093125; doi:10.3325/cmj.2025.66.100)

SUPPLEMENTAL FIGURE 2. Distribution of patients with acute lymphoblastic leukemia (ALL) in the entire cohort according to immunophenotype (BCP-ALL *vs*. T-ALL) and age.
Abbreviations: BCP – B-cell precursor.

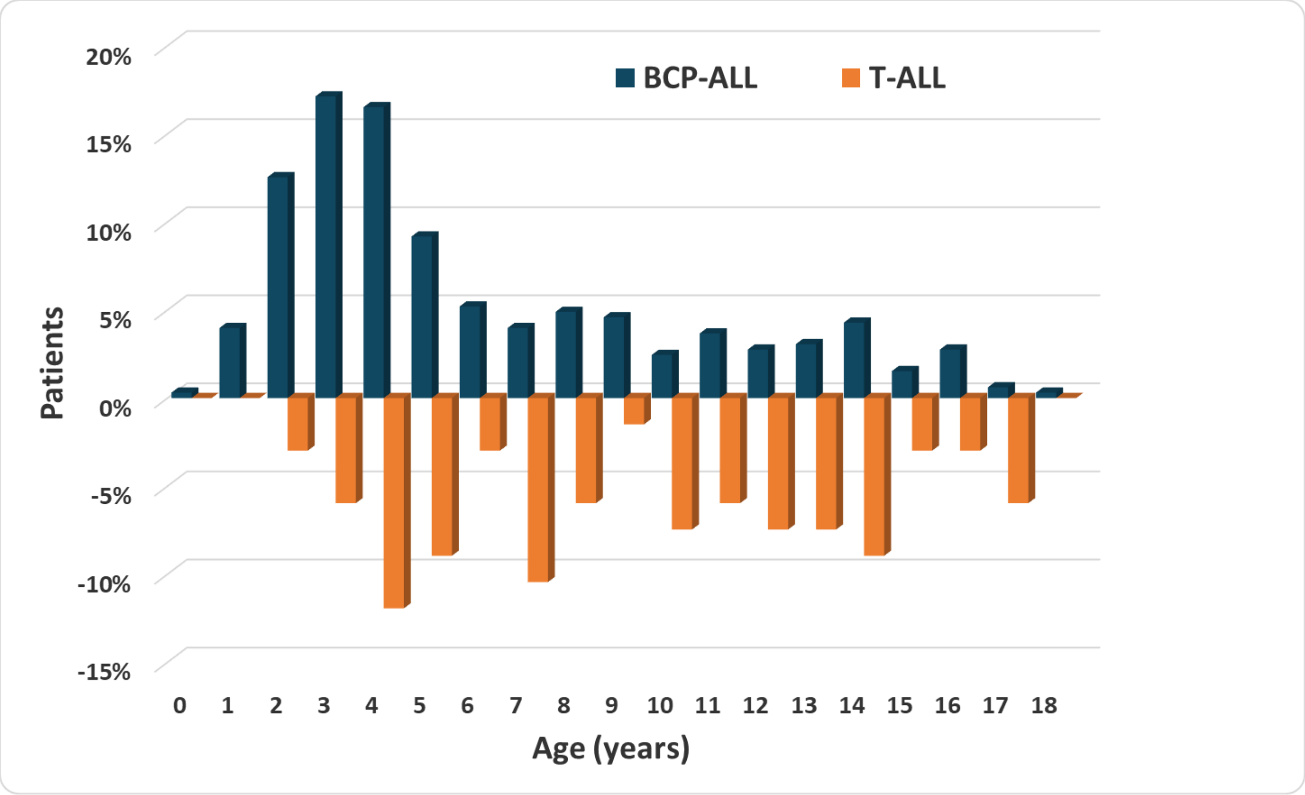

Supplement: Supplemental Figure 2 [file CroatMedJ_66_s002.pdf]
