# Supplementary material for: Disease characteristics and outcomes of Croatian pediatric patients with acute lymphoblastic leukemia: pretreatment immunophenotypic predictors of high bone marrow minimal residual disease on day 15 of treatment
Source: Croat Med J. 2025 Apr;66(2):100–14. doi: 10.3325/cmj.2025.66.100 (PMC12093125; doi:10.3325/cmj.2025.66.100)
Supplement: Supplemental Figure 3 [file CroatMedJ_66_s003.pdf]

SUPPLEMENTAL FIGURE 3. Kaplan-Meier curves for event-free survival (EFS) and overall survival (OS) according to risk groups in non-infant patients (aged 1–18 years): (**A, B**) ALL IC-BFM 2002 and (**C, D**) ALL IC-BFM 2009 protocols. Abbreviations: HR – high risk; IR – intermediate risk; SR – standard risk.

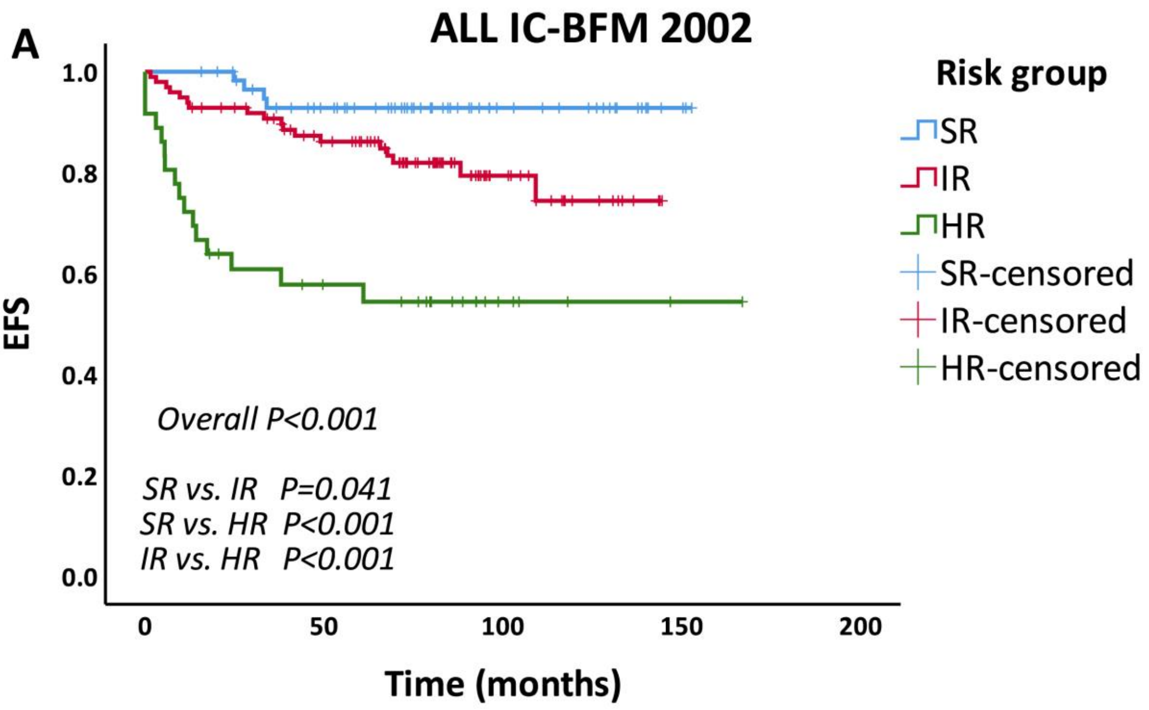

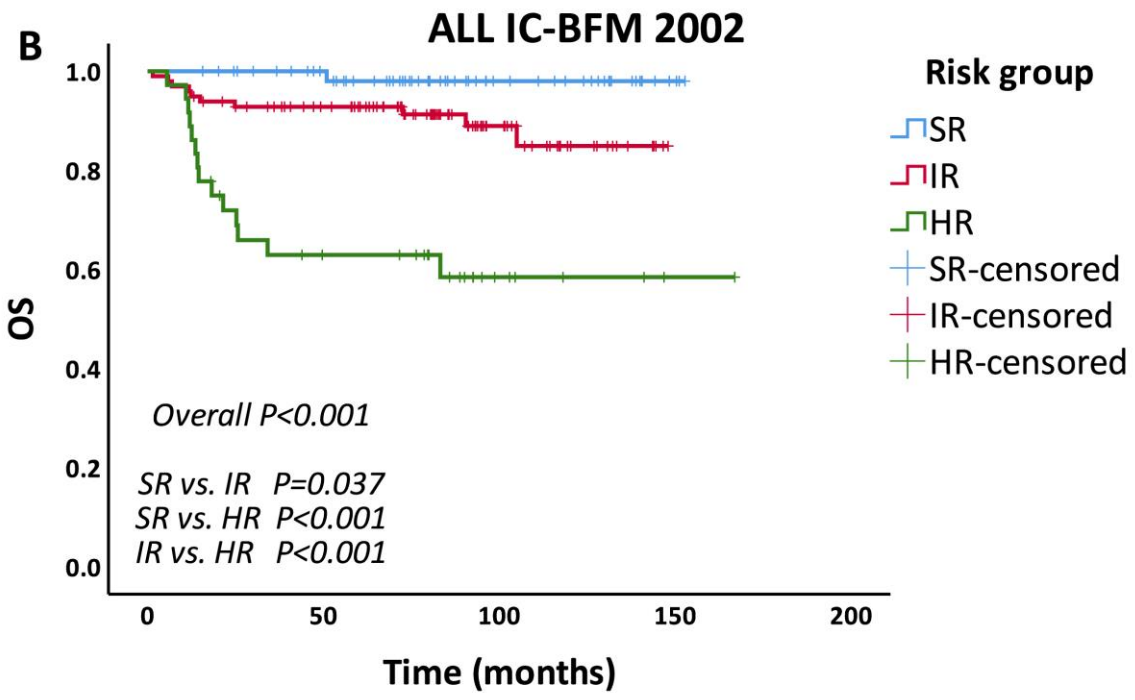

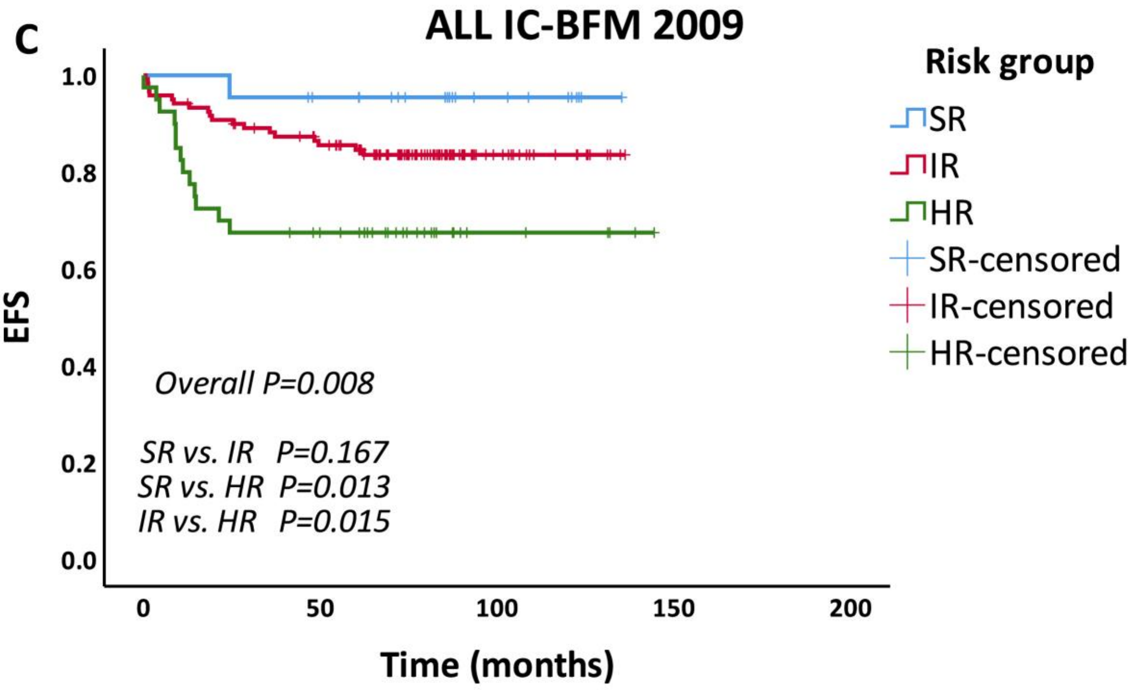
C
ALL IC-BFM 2009
Risk group
SR
IR
HR
SR-censored
IR-censored
HR-censored
EFS
1.0
0.8
0.6
0.4
0.2
0.0
0
50
100
150
200
Time (months)
Overall P=0.008
SR vs. IR P=0.167
SR vs. HR P=0.013
IR vs. HR P=0.015

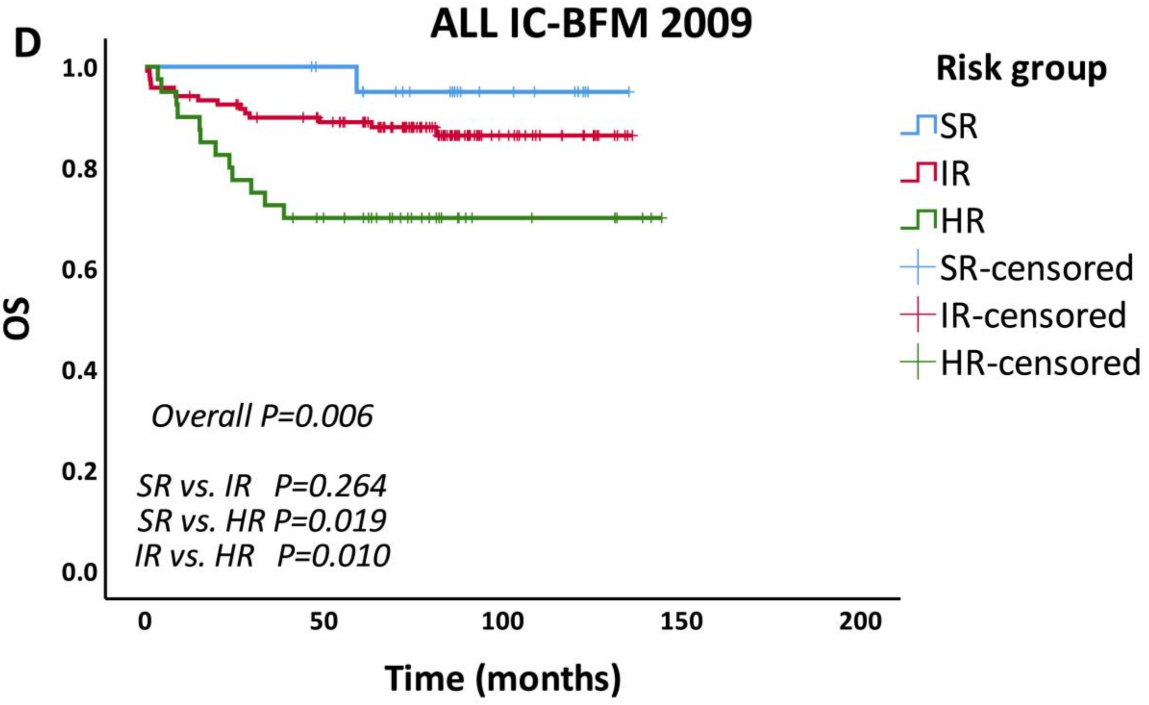
D
ALL IC-BFM 2009
Risk group
SR
IR
HR
SR-censored
IR-censored
HR-censored
OS
1.0
0.8
0.6
0.4
0.2
0.0
0
50
100
150
200
Time (months)
Overall P=0.006
SR vs. IR P=0.264
SR vs. HR P=0.019
IR vs. HR P=0.010
