# Supplementary material for: Disease characteristics and outcomes of Croatian pediatric patients with acute lymphoblastic leukemia: pretreatment immunophenotypic predictors of high bone marrow minimal residual disease on day 15 of treatment
Source: Croat Med J. 2025 Apr;66(2):100–14. doi: 10.3325/cmj.2025.66.100 (PMC12093125; doi:10.3325/cmj.2025.66.100)

SUPPLEMENTAL FIGURE 4. Cumulative incidence of relapse (CIR) according to risk groups in non-infant patients (aged 1–18 years): (**A**) ALL IC-BFM 2002 and (**B**) ALL IC-BFM 2009 protocols. Abbreviations: HR – high risk; IR – intermediate risk; SR – standard risk.

A

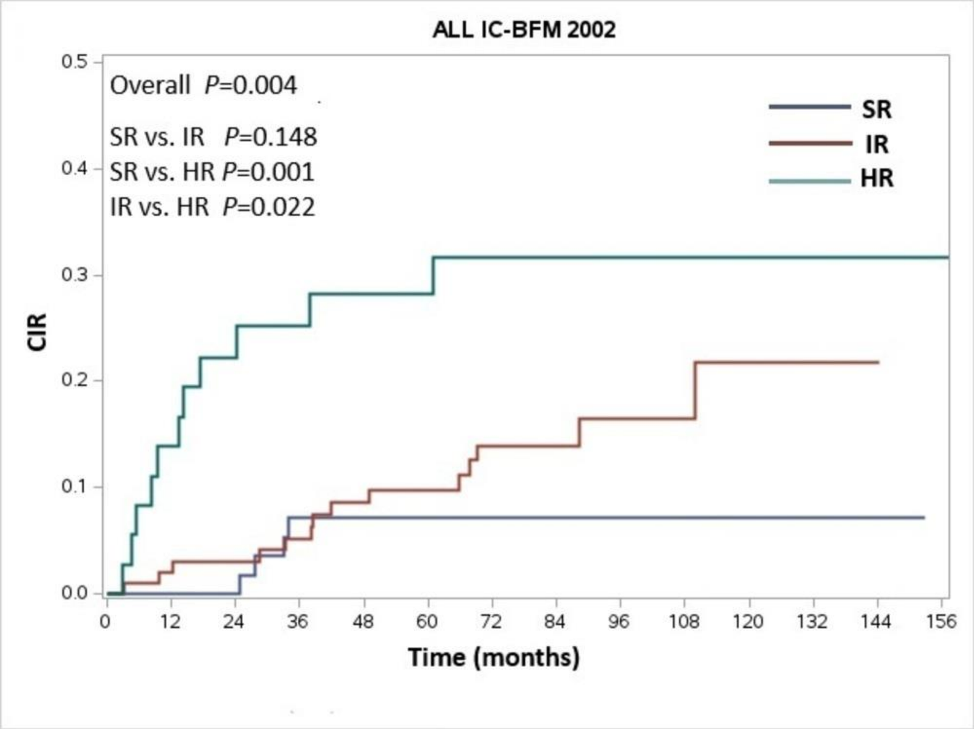

B

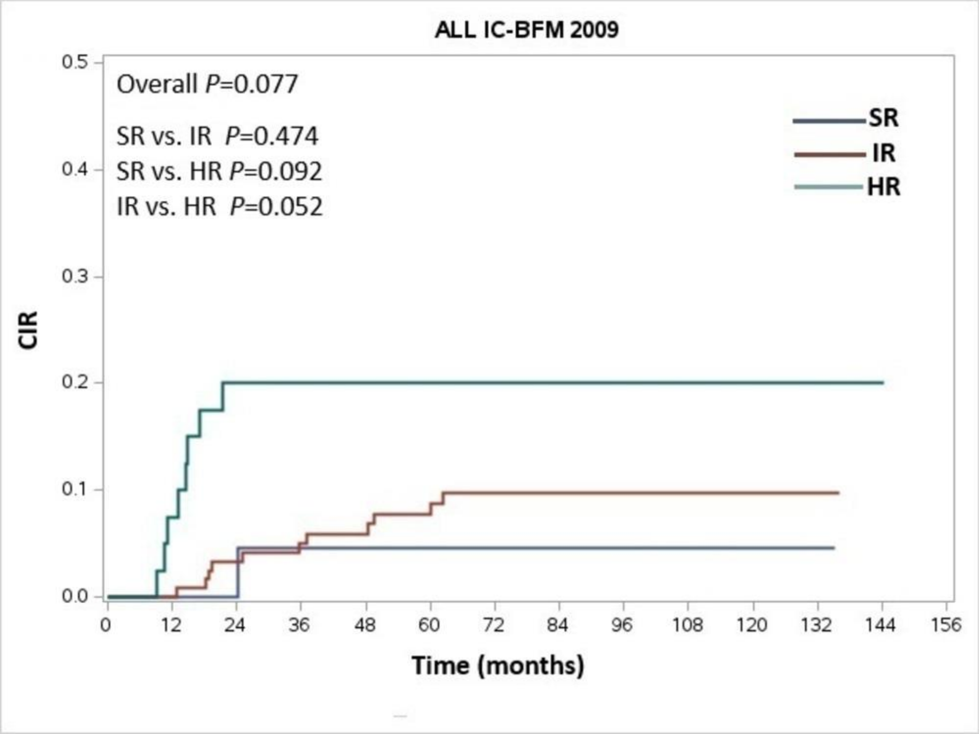

Supplement: Supplemental Figure 4 [file CroatMedJ_66_s004.pdf]
