# Supplementary material for: Disease characteristics and outcomes of Croatian pediatric patients with acute lymphoblastic leukemia: pretreatment immunophenotypic predictors of high bone marrow minimal residual disease on day 15 of treatment
Source: Croat Med J. 2025 Apr;66(2):100–14. doi: 10.3325/cmj.2025.66.100 (PMC12093125; doi:10.3325/cmj.2025.66.100)

SUPPLEMENTAL FIGURE 5. ROC curve illustrating the discriminatory ability of the combined model (WBC ≥20×10^9/L,CD13^{strong}, and CD34^{strong}) in predicting day 15 FCM-MRD ≥10% in non-infant patients (aged 1–18 years): (**A**) the entire BCP-ALL cohort (AUC 0.728; 95% CI 0.647–0.809) and (**B**) BCP-ALL patients after excluding the poor genetic prognostic group (AUC 0.734; 95% CI 0.651–0.817). Abbreviations: AUC – area under the curve; CI – confidence interval; FCM-MRD – flow cytometry–based minimal residual disease; ROC – receiver operating characteristic; WBC – white blood cells.

A

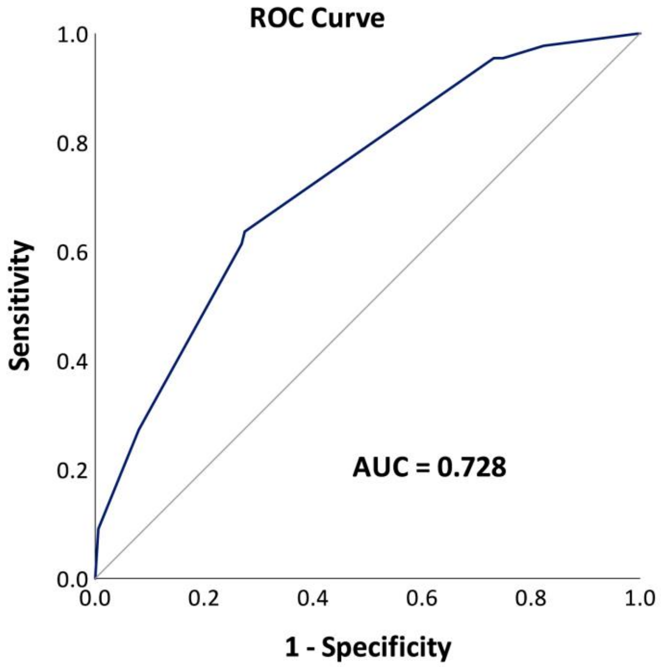

B

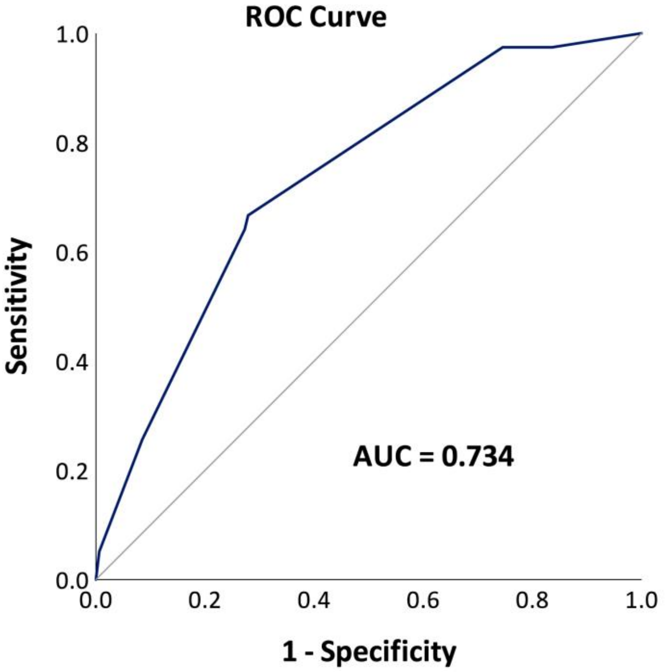

Supplement: Supplemental Figure 5 [file CroatMedJ_66_s005.pdf]
