# Supplementary material for: Disease characteristics and outcomes of Croatian pediatric patients with acute lymphoblastic leukemia: pretreatment immunophenotypic predictors of high bone marrow minimal residual disease on day 15 of treatment
Source: Croat Med J. 2025 Apr;66(2):100–14. doi: 10.3325/cmj.2025.66.100 (PMC12093125; doi:10.3325/cmj.2025.66.100)
Supplement: Supplemental Methods [file CroatMedJ_66_s006.pdf]

Supplemental Methods

Patients and treatment protocols

A retrospective analysis was performed based on data from 393 consecutive, unselected patients aged ≤18 years with *de novo* ALL diagnosed between February 2003 and April 2017 at four Croatian pediatric hemato-oncology centers. The diagnosis and classification of ALL were based on morphology, cytochemistry, immunophenotyping, karyotyping, and molecular analysis of leukemic blasts in the bone marrow samples. Two patients continued treatment abroad, and 12 were infants enrolled in the INTERFANT 99/06 protocols. Patients aged >1 year were treated according to one of two subsequent protocols: ALL IC-BFM 2002 (n=196; 49.9%) or ALL IC-BFM 2009 (n=183; 46.6%), depending on the time of diagnosis. For the FCM-MRD studies, only non-infant patients treated according to ALL IC-BFM 2002 and 2009 protocols were analyzed using pooled data since the treatment scheme for the first 15 days of remission induction remained the same. Patients were stratified into standard- (SR), intermediate- (IR), and high-risk (HR) groups based on initial age, white blood cell (WBC) count, genetic aberrations, prednisone response, bone marrow morphology on days 15 and 33 for ALL IC-BFM 2002, and additionally, day 15 FCM-MRD levels in bone marrow for ALL IC-BFM 2009 protocol (1,2). Written informed consent was obtained from the parents or legal guardians of the participants prior to the initiation of treatment. The study was approved by the Ethics Committee of the University Hospital Center Zagreb and the Ethics Committee of the Faculty of Pharmacy and Biochemistry, University of Zagreb.

Immunophenotypic analysis

Flow cytometry immunophenotyping of bone marrow aspirates at diagnosis and during follow-up for all patients was performed centrally in the Croatian Reference Centre for Immunodiagnostics of Hematological and Immunological Diseases, University Hospital Centre Zagreb. Diagnostic bone marrow samples were collected before treatment and processed within 24 hours. Briefly, bone marrow samples were subjected to red blood cell lysis using ammonium chloride solution and then washed and stained with three- or four-color panels of fluorochrome-conjugated monoclonal antibodies.

For staining of intracytoplasmic antigens, cells were permeabilized and fixed using the IntraStain kit (DAKO, Glostrup, Denmark) according to the manufacturer's instructions. Antibody clones together with conjugated fluorochromes routinely used for diagnostic immunophenotyping were: CD1a-PE (clone NA1/34), CD2-FITC (clone MT910), CD10-FITC and CD10-PE (clone SS2/36), CD13-PE (clone WM-47), CD33-PE (clone WM-54), CD79a-PE (clone HM57), MPO-PE (clone MPO-7), lysozyme-FITC (clone EC3.2.1.17), TdT-FITC (clone HT-6), IgM-FITC (clone F0058) (DAKO, Glostrup, Denmark); CD3-PE, CD3-PerCP and CD3-APC (clone SK7), CD4-FITC and CD4-PE (SK3), CD5-FITC and CD5-PerCP-Cy5.5 (clone L17F12), CD7-PE (clone M-T701), CD8-FITC and CD8-PE (clone SK1), CD19-APC (clone SJ25C1), CD20-FITC (clone L27), CD34-PE, CD34-PerCP-Cy5.5 (clone 8G12), CD45-PerCP (clone 2D1), CD117-PE (clone 104D2), HLADR-PerCP (clone L243) (BD Biosciences, San Jose, CA, USA); and CD58-FITC (clone AICD58) (Beckman Coulter, Brea, CA, SAD).

Acquisition, data collection, and analysis were done using a FacsCalibur flow cytometer and CellQuest Pro^{TM} software (both from BD Biosciences, San Jose, CA, USA). The instrument performance was monitored daily by assessing the expression of normal peripheral blood T-lymphocytes labeled with CD8-FITC/CD4-PE/CD45-PerCP/CD3-APC and weakly using CaliBRITE^{TM} beads and FACSComp software (both from BD Biosciences, San Jose, CA, USA). The classification of ALL was performed according to the EGIL recommendations, using a threshold of 20% to define positivity for surface and 10% for cytoplasmic markers (3).

In this study, we reanalyzed the raw FCM data, and the LAIPs of leukemic blasts at diagnosis were determined and reported for each antigen according to AIEOP-BFM Consensus Guidelines 2016 for ALL immunophenotyping (4). Briefly, the leukemic blast population was gated as a distinct cluster of events according to the light scatter characteristics and CD45 expression. Percentage positivity and antigen expression levels were determined by comparing the fluorescence shift and distribution pattern of the leukemic population to those of the appropriate residual normal cells, which served as internal positive or negative control cell populations. For most analyzed antigens, mature normal cross-lineage lymphocytes lacking the examined antigen were used as negative controls, while normal lymphoid or myeloid subpopulations expressing the examined antigen served as positive internal controls. Hematogone populations in non-ALL bone marrow samples, assayed within a week before or after the ALL specimens, were used as positive controls in cases where internal control

hematogones were absent. In this way, the expression levels of individual markers were categorized into several basic semi-quantitative immunophenotypic categories as being negative/weak [dim or partially positive in <50% of blasts (PP1)], or strong positive [medium, partially positive in ≥50% blasts (PP2), bright, or heterogeneous]. The expression is generally referred to as "weak" if ≥20% but <50% of the blast population expresses the respective antigen and as "strong" if the positive cell population is ≥50% (4).

Cases that fulfilled the 2017 WHO classification criteria for acute leukemias of ambiguous lineage (mixed phenotype acute leukemia) (5) and Burkitt's lymphoma/leukemia were excluded from the study.

Minimal residual disease assessment by flow cytometry

FCM-MRD was measured in the bone marrow on day 15 of induction therapy after 14 days of prednisone, vincristine, daunorubicin, asparaginase, and intrathecal methotrexate. All samples were processed according to stain/lyse protocol, using BD FACS™ Lysing Solution and analyzed using a FacsCalibur flow cytometer and Paint-a-Gate™ software (all from BD Biosciences, San Jose, CA, USA), as previously described (6,7). LAIPs of leukemic lymphoblasts were identified at diagnosis as deviations from normal lymphocyte differentiation/maturation patterns defined by previously published immunophenotypes of hematogones (8). For this purpose, the following monoclonal fluorochrome-conjugated antibody combinations were used: 1) SYTO16/CD10-PE/CD45-PerCP/CD19-APC; 2) CD20-FITC/CD10-PE/CD34-PerCP-Cy5.5/CD19-APC; 3) CD58-FITC/CD10-PE/CD45-PerCP/CD19-APC; and 4) CD10+20-FITC/CD38-PE/CD45-PerCP/CD19-APC for BCP-ALL; and 1) SYTO16/CD7-PE/CD45-PerCP/CD3-APC; 2) CD99-FITC/CD7-PE/CD5-PerCP-Cy5.5/CD3-APC; 3) TdT-FITC/CD7-PE/CD3-PerCP/citCD3-APC; and 4) CD4-FITC/CD8-PE/CD45-PerCP/CD3-APC for T-ALL. The same panels were used during follow-up for FCM-MRD detection. The presence of nonnucleated cells (platelets, red cell fragments, and debris) and hemodilution were corrected using the cell-permeant nucleic acid-binding dye SYTO16. A total of 300,000 nucleated (SYTO16+) events were acquired. Samples with significant hemodilution, defined as having less than 2% erythroblasts (SYTO16+CD45-lineage-) or less than 50% SYTO16+ events, were excluded from patient stratification. FCM-MRD is quantified as the percentage of leukemic cells among nucleated (SYTO16+) cells.

Statistical analysis

Differences between patient groups were analyzed using Pearson's chi-square or Fisher's exact test with Monte Carlo simulation for categorical variables, followed by a post hoc test with Bonferroni correction for multiple comparisons. A nonparametric Mann-Whitney U test was used to compare continuous variables. The primary endpoint of the study was the day 15 FCM-MRD in bone marrow, categorized as a dichotomous variable (FCM-MRD <10% vs. FCM-MRD ≥10%).

Univariate and multivariate logistic regression using the enter method was conducted to identify LAIP and other independent clinical and biological variables at diagnosis that predicted FCM-MRD ≥10%. Variables with $P<0.05$ in the univariate analysis were entered into the multivariate model. Associations between FCM-MRD and assessed variables were expressed as odds ratios (OR) with 95% confidence intervals (CI). Receiver operating characteristic (ROC) curves and the area under the curve (AUC) were used to assess the discriminatory ability of predictors.

The Kaplan-Meier method with log-rank tests for group comparisons was used to estimate survival rates. Event-free survival (EFS) was calculated from the date of diagnosis to the first event (relapse, resistance, second malignant neoplasm, or death). Overall survival (OS) was calculated from the date of diagnosis to all-cause death. For both analyses, observation time was censored at the last follow-up date if no event had occurred. The cumulative incidence of relapse (CIR) was calculated using the method of Kalbfleisch and Prentice and was compared between groups using Gray's test, considering death before relapse as a competing risk. Patients' follow-up data were updated in June 2023.

Two-tailed $P<0.05$ was considered statistically significant. Statistical analyses were performed using SPSS statistical software, version 26 (IBM, *Armonk, NY,* USA), and SAS OnDemand for Academics (SAS Institute Inc., Cary, NC, USA).

References

1 Stary J, Zimmermann M, Campbell M, Castillo L, Dibar E, Donska S, et al. Intensive chemotherapy for childhood acute lymphoblastic leukemia: results of the randomized intercontinental trial ALL IC-BFM 2002. J Clin Oncol. 2014;32(3):174–84.

2 Campbell M, Kiss C, Zimmermann M, Riccheri C, Kowalczyk J, Felice MS, et al. Childhood Acute Lymphoblastic Leukemia: Results of the Randomized Acute Lymphoblastic Leukemia Intercontinental-Berlin-Frankfurt-Münster 2009 Trial. J Clin Oncol. 2023;41(19):3499–511.

3 Béné MC, Castoldi G, Knapp W, Ludwig WD, Matutes E, Orfao A, et al. Proposals for the immunological classification of acute leukemias. European Group for the Immunological Characterization of Leukemias (EGIL). Leukemia. 1995;9(10):1783–6.

4 Dworzak MN, Buldini B, Gaipa G, Ratei R, Hrusak O, Luria D, et al. International-BFM-FLOW-network. AIEOP-BFM consensus guidelines 2016 for flow cytometric immunophenotyping of Pediatric acute lymphoblastic leukemia. Cytometry B Clin Cytom. 2018;94(1):82–93.

5 Swerdlow SH, Campo E, Harris NL, Jaffe ES, Pileri SA, Stein H, Thiele J. WHO Classification of Tumours of Haematopoietic and Lymphoid Tissues. Lyon, France: IARC Press;2017.

6 Mejstríková E, Fronková E, Kalina T, Omelka M, Batinic D, Dubravcic K, et al. Detection of residual B precursor lymphoblastic leukemia by uniform gating flow cytometry. Pediatr Blood Cancer. 2010;54(1):62–70.

7 Maurer-Granofszky M, Schumich A, Buldini B, Gaipa G, Kappelmayer J, Mejstrikova E, et al. An Extensive Quality Control and Quality Assurance (QC/QA) Program Significantly Improves Inter-Laboratory Concordance Rates of Flow-Cytometric Minimal Residual Disease Assessment in Acute Lymphoblastic Leukemia: An I-BFM-FLOW-Network Report. Cancers (Basel). 2021;13(23):6148.

8 Lucio P, Gaipa G, van Lochem EG, van Wering ER, Porwit-MacDonald A, Faria T, et al. BIOMED-I concerted action report: flow cytometric immunophenotyping of precursor B-ALL with standardized triple-stainings. BIOMED-1 Concerted Action Investigation of Minimal Residual Disease in Acute Leukemia: International Standardization and Clinical Evaluation. Leukemia 2001;15(8):1185–92.
