# Supplementary material for: Disease characteristics and outcomes of Croatian pediatric patients with acute lymphoblastic leukemia: pretreatment immunophenotypic predictors of high bone marrow minimal residual disease on day 15 of treatment
Source: Croat Med J. 2025 Apr;66(2):100–14. doi: 10.3325/cmj.2025.66.100 (PMC12093125; doi:10.3325/cmj.2025.66.100)
Supplement: Supplemental Table 1 [file CroatMedJ_66_s007.pdf]

SUPPLEMENTAL TABLE 1. Clinical and biological features according to EGIL subtypes of BCP-ALL*

	Total	***BCP-ALL – EGIL subtypes***			***P*†**
		Pro-B (B-I)	***Common (B-II)***	***Pre-B (B-III)***	
n (%)	326 (100.0)	*15 (4.6)*	*235 (72.1)*	*76 (23.3)*	
	n (%)	n (%)	n (%)	n (%)	
Sex					0.201
Male	193 (59.2)	10 (66.7)	132 (56.2)	51 (67.1)	
Female	133 (40.8)	5 (33.3)	103 (43.8)	25 (32.9)	
***Age** (years)*					<0.001
<1	12 (3.7)	8 (53.3)	1 (0.4)	3 (3.9)	
≥1 – <6	193 (59.2)	2 (13.3)	145 (61.7)	46 (60.5)	
≥6 – <10	49 (15.0)	2 (13.3)	36 (15.3)	11 (14.5)	
≥10 – <16	64 (19.6)	3 (20.0)	47 (20.0)	14 (18.4)	
≥16 – <18	8 (2.5)	0 (0.0)	6 (2.6)	2 (2.6)	
***WBC count** ($\times10^9$/L)*					0.001
<20	222 (69.4)	2 (16.7)	167 (72.0)	53 (69.7)	
≥20	98 (30.6)	10 (83.3)	65 (28.0)	23 (30.3)	
No information	6	3	3	0	
CNS status					0.001
CNS1	299 (93.1)	8 (61.5)	219 (94.4)	72 (94.7)	
CNS2	16 (5.0)	2 (15.4)	11 (4.7)	3 (3.9)	
CNS3	6 (1.9)	3 (23.1)	2 (0.9)	1 (1.3)	
No information	5	2	3	0	
Splenomegaly					0.946
No	166 (52.5)	6 (50.0)	120 (52.2)	40 (54.1)	
Yes	150 (47.5)	6 (50.0)	110 (47.8)	34 (45.9)	
No information	10	3	5	2	
Hepatomegaly					0.591
No	126 (39.9)	3 (25.0)	94 (40.9)	29 (39.2)	
Yes	190 (60.1)	9 (75.0)	136 (59.1)	45 (60.8)	
No information	10	3	5	2	
Mediastinal mass					1.000
No	309 (99.0)	11 (100.0)	225 (98.7)	73 (100.0)	
Yes	3 (1.0)	0 (0.0)	3 (1.3)	0 (0.0)	
No information	14	4	7	3	
Genetic prognostic groups					<0.001
Favorable	104 (32.5)	0 (0.0)	86 (37.4)	18 (24.0)	
Intermediate	188 (58.8)	3 (20.0)	133 (57.8)	52 (69.3)	
Poor	28 (8.8)	12 (80.0)	11 (4.8)	5 (6.7)	
No information	6	0	5	1	

*Abbreviations: BCP – B-cell precursor; CNS – central nervous system; EGIL – European group for immunological classification of leukemias; WBC – white blood cells.

†χ^2 or Monte Carlo simulated Fisher's exact test comparing EGIL groups; patients with no information were excluded from the test.
