# Supplementary material for: Disease characteristics and outcomes of Croatian pediatric patients with acute lymphoblastic leukemia: pretreatment immunophenotypic predictors of high bone marrow minimal residual disease on day 15 of treatment
Source: Croat Med J. 2025 Apr;66(2):100–14. doi: 10.3325/cmj.2025.66.100 (PMC12093125; doi:10.3325/cmj.2025.66.100)
Supplement: Supplemental Table 2 [file CroatMedJ_66_s008.pdf]

SUPPLEMENTAL TABLE 2. Clinical and biological features according to EGIL subtypes of T-ALL*

	**Total**	***T-ALL – EGIL subtypes***					***P*†**
		Pro-T (T-I)	***Pre-T (T-II)***	***Cortical T (T-III)***	***Mature T (T-IV)***	***Unclassified T‡***	
n (%)	67 (100.0)	*4 (6.0)*	*16 (23.9)*	*30 (44.7)*	*12 (17.9)*	*5 (7.5)*	
	n (%)	n (%)	n (%)	n (%)	n (%)	n (%)	
Sex							0.681
Male	48 (71.6)	3 (75.0)	12 (75.0)	23 (76.7)	7 (58.3)	3 (60.0)	
Female	19 (28.4)	1 (25.0)	4 (25.0)	7 (23.3)	5 (41.7)	2 (40.0)	
Age *(years)*							
<1	0 (0.0)	0 (0.0)	0 (0.0)	0 (0.0)	0 (0.0)	0 (0.0)	0.067
≥1 – <6	21 (31.3)	0 (0.0)	6 (37.5)	13 (43.3)	2 (16.7)	0 (0.0)	
≥6 – <10	13 (19.4)	0 (0.0)	1 (6.3)	5 (16.7)	5 (41.7)	2 (40.0)	
≥10 – <16	26 (38.8)	3 (75.0)	5 (31.3)	11 (36.7)	4 (33.3)	3 (60.0)	
≥16 – <18	7 (10.4)	1 (25.0)	4 (25.0)	1 (3.3)	1 (8.3)	0 (0.0)	
WBC count *($\times 10^9$/L)*							0.635
<20	12 (18.2)	1 (25.0)	2 (12.5)	5 (16.7)	3 (27.3)	1 (20.0)	
≥20	54 (81.8)	3 (75.0)	14 (87.5)	25 (83.3)	8 (72.7)	4 (80.0)	
No information	1	0	0	0	1	0	
CNS status							0.829
CNS1	56 (87.5)	4 (100.0)	12 (80.0)	26 (89.7)	10 (90.9)	4 (80.0)	
CNS2	4 (6.3)	0 (0.0)	2 (13.3)	1 (3.4)	0 (0.0)	1 (20.0)	
CNS3	4 (6.3)	0 (0.0)	1 (6.7)	2 (6.9)	1 (9.1)	0 (0.0)	
No information	3	0	1	1	1	0	
Splenomegaly							0.267
No	22 (33.8)	3 (75.0)	5 (33.3)	11 (36.7)	2 (18.2)	1 (20.0)	
Yes	43 (66.2)	1 (25.0)	10 (66.7)	19 (63.3)	9 (81.8)	4 (80.0)	
No information	2	0	1	0	1	0	
Hepatomegaly							0.157
No	24 (36.9)	1 (25.0)	9 (60.0)	8 (26.7)	5 (45.5)	1 (20.0)	
Yes	41 (63.1)	3 (75.0)	6 (40.0)	22 (73.3)	6 (54.5)	4 (80.0)	
No information	2	0	1	0	1	0	
Mediastinal mass							0.039
No	31 (48.4)	2 (50.0)	9 (64.3)	8 (26.7)	7 (63.6)	5 (100.0)	
Yes	33 (51.6)	2 (50.0)	5 (35.7)	22 (73.3)	4 (36.4)	0 (0.0)	
No information	3	0	2	0	1	0	
Genetic prognostic groups							0.551
Favorable	1 (1.6)	0 (0.0)	1 (6.3)	0 (0.0)	0 (0.0)	0 (0.0)	
Intermediate	57 (93.4)	3 (100.0)	15 (93.8)	26 (92.9)	9 (90.0)	4 (100.0)	
Poor	3 (4.9)	0 (0.0)	0 (0.0)	2 (7.1)	1 (10.3)	0 (0.0)	
No information	6	1	0	2	2	1	

*Abbreviations: CNS – central nervous system; EGIL – European group for immunological classification of leukemias; WBC – white blood cells.

†χ^2 or Monte Carlo simulated Fisher's exact test comparing EGIL groups, excluding unclassified T and patients without information.

‡EGIL classification could not be performed due to incomplete immunophenotypic data.
