# Supplementary material for: Disease characteristics and outcomes of Croatian pediatric patients with acute lymphoblastic leukemia: pretreatment immunophenotypic predictors of high bone marrow minimal residual disease on day 15 of treatment
Source: Croat Med J. 2025 Apr;66(2):100–14. doi: 10.3325/cmj.2025.66.100 (PMC12093125; doi:10.3325/cmj.2025.66.100)
Supplement: Supplemental Table 3 [file CroatMedJ_66_s009.pdf]

SUPPLEMENTAL TABLE 3. Early response to treatment between BCP-ALL and T-ALL in patients treated with ALL IC-BFM 2002/2009 protocols*

ALL IC-BFM 2002/2009	*Total*	*BCP-ALL*	*T-ALL*	*P*†
n (%)	379 (100.0)	312 (82.3)	67 (17.7)	
Prednisone response day 8				<0.001
GPR	344 (93.2)	293 (96.4)	51 (78.5)	
PPR	25 (6.8)	11 (3.6)	14 (21.5)	
No information	10	8	2	
BM morphology day 15				0.007
M1	266 (77.6)	220 (77.7)	46 (76.7)	
M2	54 (15.7)	49 (17.3)	5 (8.3)	
M3	23 (6.7)	14 (4.9)	9 (15.0)	
No information	36	29	7	
FCM-MRD day 15				0.084
MRD <10%	239 (77.9)	201 (79.8)	38 (69.1)	
MRD *≥10%*	68 (22.1)	51 (20.2)	17 (30.9)	
No information/inadequate sample	72	60	12	

*Abbreviations: BM – bone marrow; FCM – flow cytometry; GPR – good prednisone response (<1000 blasts/µL in peripheral blood on day 8); M1 – <5% blasts; M2 – 5–25% blasts; M3 – ≥25% blasts in bone marrow; MRD – minimal residual disease; PPR – poor prednisone response (≥1000 blasts/µL in peripheral blood on day 8).
†χ^2 test comparing patient groups; patients without data or with an inadequate sample were excluded from the test.
