# Supplementary material for: Disease characteristics and outcomes of Croatian pediatric patients with acute lymphoblastic leukemia: pretreatment immunophenotypic predictors of high bone marrow minimal residual disease on day 15 of treatment
Source: Croat Med J. 2025 Apr;66(2):100–14. doi: 10.3325/cmj.2025.66.100 (PMC12093125; doi:10.3325/cmj.2025.66.100)
Supplement: Supplemental Table 4 [file CroatMedJ_66_s010.pdf]

SUPPLEMENTAL TABLE 4. Patients' characteristics and response to treatment according to FCM-MRD levels in BCP-ALL and T-ALL in patients treated with ALL IC-BFM 2002/2009 protocols*

	BCP-ALL			***T-ALL***		
	FCM-MRD day 15			***FCM-MRD day 15***		
	MRD<10%	*MRD≥10%*	***P†***	*MRD<10%*	*MRD≥10%*	***P†***
	n (%)	n (%)		n (%)	n (%)	
Sex			0.062			0.489
Male	113 (56.2)	36 (70.6)		26 (68.4)	10 (58.8)	
Female	88 (43.8)	15 (29.4)		12 (31.6)	7 (41.2)	
Age *(years)*			0.011			0.058
<6	137 (68.2)	25 (49.0)		15 (39.5)	2 (11.8)	
≥6	64 (31.8)	26 (51.0)		23 (60.5)	15 (88.2)	
EGIL subtype			0.162			0.113
Pro-B (B-I)	1 (0.5)	1 (2.0)				
Common (B-II)	149 (74.1)	42 (82.4)				
Pre-B (B-III)	51 (25.4)	8 (15.7)				
Pro-T (T-I)				1 (2.9)	2 (11.8)	
Pre-T (T-II)				8 (23.5)	4 (23.5)	
Cortical T(T-II)				20 (58.8)	5 (29.4)	
Mature T (T-IV)				5 (14.7)	6 (35.3)	
Unclassified T				4	0	
WBC count *($\times 10^9$/L)*			0.001			0.709
<20	149 (74.5)	26 (51.0)		7 (18.4)	2 (12.5)	
≥20	51 (25.5)	25 (49.0)		31 (81.6)	14 (87.5)	
No information	1	0		0	1	
CNS status			1.000			1.000
CNS1	188 (94.0)	49 (96.1)		33 (86.8)	13 (86.7)	
CNS2	10 (5.0)	2 (3.9)		2 (5.3)	1 (6.7)	
CNS3	2 (1.0)	0 (0.0)		3 (7.9)	1 (6.7)	
No information	1	0		0	2	
Splenomegaly			0.136			0.751
No	106 (53.8)	21 (42.0)		12 (31.6)	4 (25.0)	
Yes	91 (46.2)	29 (58.0)		26 (68.4)	12 (75.0)	
No information	4	1		0	1	
Hepatomegaly			0.940			0.981
No	76 (38.6)	19 (38.0)		12 (31.6)	5 (31.3)	
Yes	121 (61.4)	31 (62.0)		26 (68.4)	11 (68.8)	
No information	4	1		0	1	

SUPPLEMENTAL TABLE 4. Continued.

	BCP-ALL			***T-ALL***		
	FCM-MRD day 15			***FCM-MRD day 15***		
	MRD<10%	*MRD≥10%*	***P†***	*MRD<10%*	*MRD≥10%*	***P†***
	n (%)	n (%)		n (%)	n (%)	
Mediastinal mass			1.000			0.743
No	192 (98.5)	50 (100.0)		18 (48.6)	7 (43.8)	
Yes	3 (1.5)	0 (0.0)		19 (51.4)	9 (56.3)	
No information	6	1		1	1	
Genetic prognostic group			0.167			0.030
Favorable	73 (36.7)	16 (32.0)		1 (3.0)	0 (0.0)	
Intermediate	117 (58.8)	28 (56.0)		32 (97.0)	13 (81.2)	
Poor	9 (4.5)	6 (12.0)		0 (0.0)	3 (18.8)	
No information	2	1		5	1	
Prednisone response day 8			0.001			<0.001
GPR	192 (98.5)	44 (86.3)		37 (97.4)	6 (37.5)	
PPR	3 (1.5)	7 (13.7)		1 (2.6)	10 (62.5)	
No information	6	0		0	1	
BM morphology day 15			<0.001			<0.001
M1	163 (89.1)	13 (26.5)		33 (91.7)	5 (35.7)	
M2/M3	20 (10.9)	36 (73.5)		3 (8.3)	9 (64.3)	
No information	18	2		2	3	
Relapse			<0.001			0.235
No	184 (92.0)	37 (72.5)		34 (89.5)	13 (76.5)	
Yes	16 (8.0)	14 (27.5)		4 (10.5)	4 (23.5)	
Death			<0.001			0.006
No	183 (91.0)	35 (68.6)		35 (92.1)	10 (58.8)	
Yes	18 (9.0)	16 (31.4)		3 (7.9)	7 (41.2)	

*Abbreviations: BM – bone marrow; FCM – flow cytometry; GPR – good response to prednisone (<1000 blasts/µL in peripheral blood on day 8); PPR – poor response to prednisone (≥1000 blasts/µL in peripheral blood on day 8); M1 – <5% blasts; M2 – 5–25% blasts; M3 – ≥25% blasts in bone marrow; MRD – minimal residual disease; WBC – white blood cells.

†χ^2 or Monte Carlo simulated Fisher's exact test comparing patient groups; patients without data or with an inadequate sample were excluded from the test.
