# Supplementary material for: Disease characteristics and outcomes of Croatian pediatric patients with acute lymphoblastic leukemia: pretreatment immunophenotypic predictors of high bone marrow minimal residual disease on day 15 of treatment
Source: Croat Med J. 2025 Apr;66(2):100–14. doi: 10.3325/cmj.2025.66.100 (PMC12093125; doi:10.3325/cmj.2025.66.100)
Supplement: Supplemental Table 5 [file CroatMedJ_66_s011.pdf]

SUPPLEMENTAL TABLE 5. Antigen expression by FCM-MRD levels in BCP-ALL patients treated with ALL IC-BFM 2002/2009 protocols*

BCP-ALL							
Antigen	***FCM-MRD day 15***			***Pooled categories***	***FCM-MRD day 15***		
	MRD<10%	*MRD≥10%*	***P†***		*MRD<10%*	*MRD≥10%*	***P†***
	n (%)	n (%)			n (%)	n (%)	
CD10			0.447				0.971
N	1 (0.5)	1 (2.0)		N/weak	3 (1.5)	1 (2.0)	
PP1	2 (1.0)	0 (0.0)					
Medium	3 (1.5)	0 (0.0)		Strong	197 (98.5)	50 (98.0)	
PP2	0 (0.0)	1 (2.0)					
Heterogeneous	12 (6.0)	2 (3.9)					
Normal	54 (27.0)	15 (29.4)					
Overexpression	128 (64.0)	32 (62.7)					
No information	1	0					
CD19			0.033				
Dim	3 (1.5)	4 (7.8)					
Normal	197 (98.5)	47 (92.2)					
No information	1	0					
CD20			0.191				0.983
N	93 (46.7)	27 (54.0)		N/weak	139 (69.8)	35 (70.0)	
Dim	42 (21.1)	5 (10.0)					
PP1	4 (2.0)	3 (6.0)					
Medium	42 (21.1)	11 (22.0)		Strong	60 (30.2)	15 (30.0)	
PP2	4 (2.0)	1 (2.0)					
Heterogeneous	3 (1.5)	2 (4.0)					
Bright	11 (5.5)	1 (2.0)					
No information	2	1					
CD34			0.012				0.001
N	25 (12.6)	0 (0.0)		N/weak	53 (26.8)	3 (5.9)	
Dim	21 (10.6)	2 (3.9)					
PP1	7 (3.5)	1 (2.0)					
Medium	78 (39.4)	27 (52.9)		Strong	145 (73.2)	48 (94.1)	
PP2	14 (7.1)	2 (3.9)					
Bright	53 (26.8)	19 (37.3)					
No information	3	0					
CD45			0.075				0.020
N	22 (12.2)	6 (12.8)		N/weak	174 (96.7)	41 (87.2)	
Dim	110 (61.1)	24 (51.1)					
Medium	42 (23.3)	11 (23.4)					
Bright	6 (3.3)	6 (12.8)		*Bright*	6 (3.3)	6 (12.8)	
No information	21	4					
CD58			1.000				
N	5 (3.9)	1 (2.7)					
Overexpression	122 (96.1)	36 (97.3)					
No information	74	14					

SUPPLEMENTAL TABLE 5. Continued.

BCP-ALL							
Antigen	***FCM-MRD day 15***			***Pooled categories***	***FCM-MRD day 15***		
	MRD<10%	*MRD≥10%*	***P†***		*MRD<10%*	*MRD≥10%*	***P†***
	n (%)	n (%)			n (%)	n (%)	
TdT			0.073				0.427
N	2 (1.0)	0 (0.0)					
Dim	18 (9.4)	3 (6.0)		*N/weak*	21 (10.9)	3 (6.0)	
PP1	1 (0.5)	0 (0.0)					
Medium	100 (52.1)	36 (72.0)					
PP2	68 (35.4)	9 (18.0)		Strong	171 (89.1)	47 (94.0)	
Bright	3 (1.6)	2 (4.0)					
No information	9	1					
CD79a			0.122				
N	1 (0.5)	0 (0.0)					
Medium	31 (15.6)	12 (23.5)					
PP2	0 (0.0)	1 (2.0)					
Bright	167 (83.9)	38 (74.5)					
No information	2	0					
CD13			0.008				0.001
N	115 (65.3)	22 (50.0)					
Dim	41 (23.3)	8 (18.2)		N/weak	158 (89.8)	31 (70.5)	
PP1	2 (1.1)	1 (2.3)					
Medium	16 (9.1)	10 (22.7)					
PP2	2 (1.1)	1 (2.3)		Strong	18 (10.2)	13 (29.5)	
Bright	0 (0.0)	2 (4.5)					
No information	25	7					
CD15			1.000				
N	167 (98.8)	41 (100.0)					
Dim	1 (0.6)	0 (0.0)					
Medium	1 (0.6)	0 (0.0)					
No information	32	10					
CD33			0.263				0.424
N	105 (59.3)	29 (63.0)					
Dim	37 (20.9)	5 (10.9)		N/weak	144 (81.4)	35 (76.1)	
PP1	2 (1.1)	1 (2.2)					
Medium	20 (11.3)	9 (19.6)					
PP2	13 (7.3)	2 (4.3)		Strong	33 (18.6)	11 (23.9)	
Bright	0 (0.0)	0 (0.0)					
No information	24	5					
CD117			1.000				1.000
N	86 (86.0)	22 (88.0)		N/weak	97 (97.0)	24 (96.0)	
Dim	11 (11.0)	2 (8.0)					
Medium	3 (3.0)	1 (4.0)		Strong	3 (3.0)	2 (4.0)	
No information	101	26					

*Abbreviations: FCM – flow cytometry; MRD – minimal residual disease; N – negative; PP1 – partially positive in <50% of blasts; PP2 – partially positive in ≥50% of blasts.

†χ^2 or Monte Carlo simulated Fisher's exact test comparing patient groups; patients without data or with an inadequate sample were excluded from the test.
