# Supplementary material for: Disease characteristics and outcomes of Croatian pediatric patients with acute lymphoblastic leukemia: pretreatment immunophenotypic predictors of high bone marrow minimal residual disease on day 15 of treatment
Source: Croat Med J. 2025 Apr;66(2):100–14. doi: 10.3325/cmj.2025.66.100 (PMC12093125; doi:10.3325/cmj.2025.66.100)
Supplement: Supplemental Table 6 [file CroatMedJ_66_s012.pdf]

SUPPLEMENTAL TABLE 6. Antigen expression by FCM-MRD levels in T-ALL patients treated with ALL IC-BFM 2002/2009 protocols*

T-ALL							
Antigen	***FCM-MRD day 15***			***Pooled categories***	***FCM-MRD day 15***		
	MRD<10%	*MRD≥10%*	***P†***		*MRD<10%*	*MRD≥10%*	***P†***
	n (%)	n (%)			n (%)	n (%)	
CD1a			0.075				
N	16 (44.4)	12 (70.6)					
P	20 (55.6)	5 (29.4)					
No information	2	0					
CD2			0.310				
N	2 (7.7)	3 (23.1)					
P	24 (92.3)	10 (76.9)					
No information	12	4					
Cytoplasmic CD3							
P	38 (100.0)	17 (100.0)					
Membrane CD3			0.328				
N	20 (55.6)	7 (41.2)					
P	16 (44.4)	10 (58.8)					
No information	2	0					
CD4			0.108				
N	9 (25.0)	8 (47.1)					
P	27 (75.0)	9 (52.9)					
No information	2	0					
CD5			0.255				
N	1 (2.9)	2 (11.8)					
P	33 (97.1)	15 (88.2)					
No information	4	0					
CD7							
P	37 (100.0)	17 (100.0)					
No information	1	0					
CD8			0.006				
N	11 (30.6)	12 (70.6)					
P	25 (69.4)	5 (29.4)					
No information	2	0					
CD10			0.031				
N	11 (33.3)	10 (66.7)					
P	22 (66.7)	5 (33.3)					
No information	5	2					
CD13			0.598				
N	27 (84.4)	11 (73.3)					
Dim	2 (6.3)	2 (13.3)					
Medium	3 (9.4)	2 (13.3)					
No information	6	2					
CD33			0.029				0.030
N	29 (93.5)	12 (80.0)		N/weak	31 (100.0)	12 (80.0)	
Dim	2 (6.5)	0 (0.0)					
Medium	0 (0.0)	3 (20.0)		Strong	0 (0.0)	3 (20.0)	
No information	7	2					

SUPPLEMENTAL TABLE 6. Continued.

T-ALL							
Antigens	**FCM-MRD day 15**			***Pooled categories***	**FCM-MRD day 15**		
	MRD<10%	*MRD≥10%*	***P†***		*MRD<10%*	*MRD≥10%*	***P†***
	n (%)	n (%)			n (%)	n (%)	
CD34			0.004				
N	26 (78.8)	6 (37.5)					
P	7 (21.2)	10 (62.5)					
No information	5	1					
CD45			0.664				0.338
N	0 (0.0)	0 (0.0)					
Dim	1 (2.9)	0 (0.0)		N/weak	23 (67.6)	8 (53.3)	
Medium	22 (64.7)	8 (53.3)					
Bright	11 (32.4)	7 (46.7)		*Bright*	11 (32.4)	7 (46.7)	
No information	4	2					
CD79a			0.929				
N	14 (37.8)	6 (35.3)					
Dim	16 (43.2)	9 (52.9)					
Medium	4 (10.8)	1 (5.9)					
Bright	3 (8.1)	1 (5.9)					
No information	1	0					
TdT			0.216				0.021
N	3 (7.9)	2 (11.8)		N/weak	14 (36.8)	12 (70.6)	
Dim	11 (28.9)	10 (58.8)					
Medium	15 (39.5)	4 (23.5)					
PP2	5 (13.2)	1 (5.9)		Strong	24 (63.2)	5 (29.4)	
Bright	4 (10.5)	0 (0.0)					
CD117			0.038				0.020
N	28 (87.5)	9 (64.3)		N/weak	30 (93.8)	9 (64.3)	
Dim	2 (6.3)	0 (0.0)					
Medium	2 (6.3)	5 (35.7)		Strong	2 (6.3)	5 (35.7)	
No information	6	3					
HLA-DR			0.652				
N	25 (89.3)	13 (81.3)					
P	3 (10.7)	3 (18.8)					
No information	10	1					

*Abbreviations: FCM – flow cytometry; MRD – minimal residual disease; N – negative; P – positive; PP1 – partially positive in <50% of blasts; PP2 – partially positive in ≥50% of blasts.

†χ^2 or Monte Carlo simulated Fisher's exact test comparing patient groups; patients without data or with an inadequate sample were excluded from the test.
