# Supplementary material for: Disease characteristics and outcomes of Croatian pediatric patients with acute lymphoblastic leukemia: pretreatment immunophenotypic predictors of high bone marrow minimal residual disease on day 15 of treatment
Source: Croat Med J. 2025 Apr;66(2):100–14. doi: 10.3325/cmj.2025.66.100 (PMC12093125; doi:10.3325/cmj.2025.66.100)
Supplement: Supplemental Table 7 [file CroatMedJ_66_s013.pdf]

SUPPLEMENTAL TABLE 7. Univariate and multivariate logistic regression analyses for predictors of high FCM-MRD (≥10%) on Day 15 in BCP-ALL patients treated with ALL IC-BFM 2002/2009 protocols, excluding poor genetic prognostic group*

Variable†	***Univariate logistic regression***			***Multivariate logistic regression***		
	OR	***95% CI***	***P***	***OR***	***95% CI***	***P***
Male gender	2.069	1.004–4.261	**0.049**	2.012	0.884–4.577	0.095
Age ≥6 years	1.897	0.972–3.702	0.061	2.050	0.925–4.544	0.077
WBC ≥20×10^9/L	2.448	1.243–4.821	**0.010**	2.887	1.283–6.496	**0.010**
EGIL subtype						
Common (B-II)	2.711	1.011–7.267	**0.047**	2.892	0.900–9.289	0.075
Pre-B (B-III)	Reference					
Genetic prognostic group						
Favorable	Reference					
Intermediate	1.092	0.553–2.156	0.800			
**Antigen**						
CD13 strong	3.230	1.378–7.572	**0.007**	3.804	1.467–9.863	**0.006**
CD20 strong	0.912	0.437–1.905	0.807			
CD33 strong	1.417	0.629–3.193	0.401			
CD34 strong	7.200	1.679–30.876	**0.008**	7.643	1.599–36.546	**0.011**
CD45 strong	2.621	0.600–11.447	0.200			
CD117 strong	1.460	0.145–14.754	0.748			
TdT N/weak	0.608	0.172–2.146	0.439			

*Abbreviations: CI – confidence interval; EGIL – European group for immunological classification of leukemias; N – negative; OR – odds ratio; WBC – white blood cells.

†Pro-B, CD10, CD15, CD19, and CD58 were excluded from the analysis due to their low frequencies after excluding the poor genetic prognostic group.
